# Functional lung avoidance planning with combined ventilation-perfusion imaging versus individual functional imaging in lung cancer radiotherapy

**DOI:** 10.1016/j.phro.2026.101118

**Published:** 2026-11-03

**Authors:** Genta Michimata, Yujiro Nakajima, Tomoki Kimura, Nobuki Imano, Shinya Ito, Yukio Fujita, Naoki Tohyama

**Affiliations:** a Department of Radiological Sciences, Komazawa University, 1-23-1 Komazawa, Setagaya-ku, Tokyo, 154-8525, Japan; b Department of Radiation Oncology, International Medical Center, Saitama Medical University, 1397-1 Yamane, Hidaka-shi, Saitama 350-1298, Japan; c Department of Radiation Oncology, Tokyo Metropolitan Cancer and Infectious Diseases Center Komagome Hospital, 3-18-22 Honkomagome, Bunkyo-ku, Tokyo 113-8677, Japan; d Department of Radiation Oncology, Kochi Medical School, Kochi University, Kohasu, Oko-cho, Nankoku-shi, Kochi 783-8505, Japan; e Department of Radiation Oncology, Graduate School of Biomedical and Health Sciences, Hiroshima University, 1-2-3 Kasumi, Minami-ku, Hiroshima 734-8553, Japan

**Keywords:** Non-small-cell lung carcinoma, Lung ventilation, Lung perfusion, Single-photon emission computed tomography, Treatment planning, Image-guided radiation therapy

## Abstract

**Background and purpose:**

Functional lung avoidance radiotherapy using four-dimensional computed tomography (4DCT) ventilation or single-photon emission computed tomography (SPECT) perfusion has been studied independently, however few studies have evaluated their combined use. The aim of this study was to examine whether integrating both modalities improved sparing of high functional lung (HFL) in non-small cell lung cancer (NSCLC).

**Materials and methods:**

Forty-seven patients with NSCLC underwent 4DCT ventilation and SPECT perfusion imaging. One patient was excluded, leaving 46 for analysis. Functional lung maps were segmented into high, middle, and low functional lung. Four volumetric-modulated arc therapy (VMAT) plans using 6 MV photons per patient were generated: anatomical (A), ventilation-guided (V), perfusion-guided (Q), and combined ventilation-perfusion-guided (VQ). Dose-volume and dose-function metrics were compared among plans. Correlations between tumor proximity to HFL and dose reduction were assessed.

**Results:**

All function-guided plans significantly reduced HFL dose compared with A plans (*p* < 0.001). Among the function-guided plans, VQ plans yielded the lowest mean lung doses (MLDs) in high ventilated lung (VQ plans, 11.5 Gy; A plans, 12.4 Gy) and high perfused lung (VQ plans, 10.0 Gy; A plans, 11.1 Gy). Function-weighted metrics, including functional MLD, were also lowest with VQ plans, indicating improved functional lung sparing. Dose reduction correlated with tumor proximity to HFL, with the strongest associations observed at planning target volume expansions between 25 and 45 mm.

**Conclusions:**

Integrating 4DCT ventilation and SPECT perfusion into radiotherapy planning improved functional lung sparing compared with anatomical or single-modality planning. Tumor proximity to functional lung influenced the magnitude of dose-volume benefits.

## Highlights

• Treatment plans were optimized using ventilation and/or perfusion imaging.
• Combined imaging lowered mean dose to high ventilated lung from 12.4 to 11.5 Gy.
• In high perfused lung, mean dose fell from 11.1 to 10.0 Gy versus anatomical plans.
• Combined plans reduced mean dose by 0.2–0.5 Gy more than single-modality plans.
• Dose reductions peaked when high functional lungs were 25–45 mm from the target.

## Graphical abstract

**Figure:**
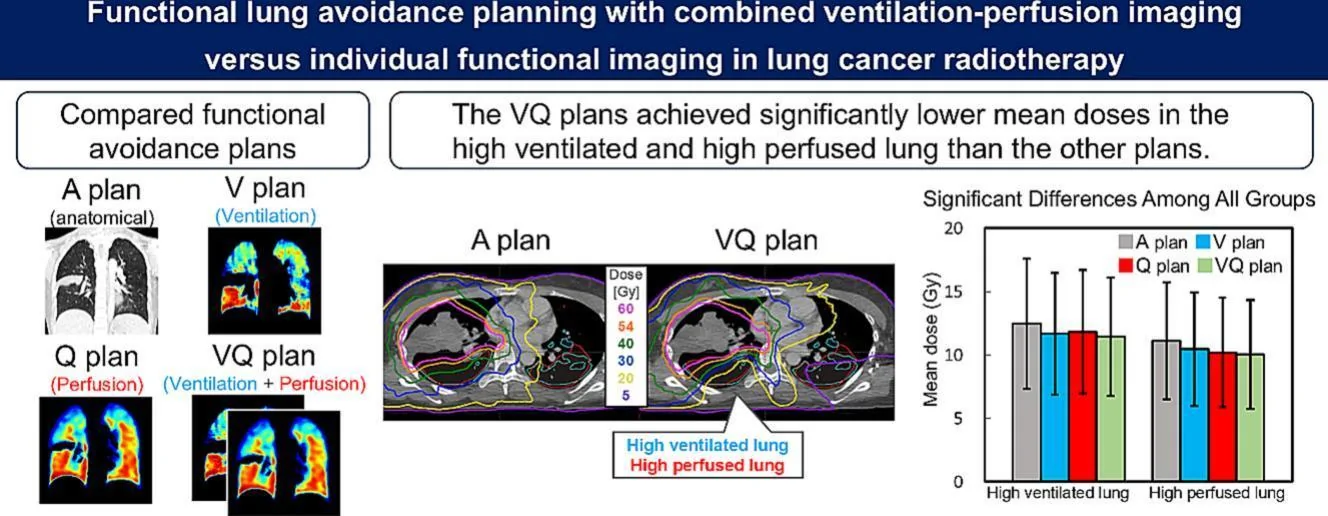

## 1 Introduction

Non-small cell lung cancer (NSCLC) is a leading cause of cancer-related mortality worldwide, and radiotherapy plays a central role in treating locally advanced disease. However, radiation pneumonitis (RP) remains a major complication that adversely affects post-treatment quality of life and prognosis, with grade ≥ 2 RP occurring in approximately 30% of patients [1]. Recently, maintenance therapy with an anti-programmed death ligand 1 (PD-L1) antibody following concurrent chemoradiotherapy has shown survival benefits, as demonstrated in the phase III, randomized, placebo-controlled PACIFIC trial involving patients with unresectable stage III NSCLC [2]. Nevertheless, RP is a common reason for the discontinuation of immune checkpoint inhibitors (ICIs), making its effective management an ongoing clinical challenge [3].

The utility of functional avoidance planning, which incorporates lung function to reduce the risk of RP, has been widely investigated. A Phase II trial using four-dimensional computed tomography (4DCT) derived ventilation images reported favorable clinical outcomes, with the incidence of RP reduced to 15% [4]. In addition to ventilation, pulmonary perfusion has also been extensively evaluated as a target for functional preservation. A meta-analysis demonstrated that incorporating either ventilation or perfusion imaging significantly reduced the radiation dose to each high functional lung (HFL) [5].

However, in patients with lung cancer, ventilation and perfusion distributions do not always coincide, resulting in ventilation-perfusion (VQ) mismatch, which poses a challenge for functional avoidance planning. Previous studies have reported that approximately 39% of lung cancer patients exhibit VQ mismatch [6]. Most prior studies have relied on either ventilation or perfusion alone; however, such approaches cannot eliminate the risk of unintentionally delivering higher doses to the other functional regions during optimization. Specifically, ventilation avoidance planning may insufficiently spare high perfused lung (HPL), whereas perfusion avoidance planning may inadequately preserve high ventilated lung (HVL).

Studies incorporating both ventilation and perfusion remain limited, and the impact of optimizing one function on the other has not been quantitatively evaluated. Previous studies have primarily focused on comparisons between ventilation-perfusion avoidance planning using gallium-68 positron emission tomography/computed tomography (^68^Ga-PET/CT) and anatomical planning [7], [8], [9], without direct comparisons to single-modality approaches such as ventilation-only or perfusion-only planning. Furthermore, the spatial distribution of lung function varies considerably among patients, and the relationship between tumor location and HFL is a critical factor in treatment planning.

The purpose of this study was to quantitatively evaluate the impact of treatment planning based on different functional imaging strategies on the planned dose distribution to functional lung regions in patients with NSCLC. In addition, the study assessed how the spatial relationship between the tumor and HFL (ventilation and perfusion) influences treatment planning outcomes.

## 2 Materials and methods

### 2.1 Study overview

This study was designed in accordance with the research design reported by Yamamoto et al., which was a planning study comparing 4DCT ventilation avoidance and anatomical planning [10]. Ventilation images derived from 4DCT and perfusion images obtained from single-photon emission computed tomography (SPECT) were converted into percentile images. Each image was segmented into three equal parts based on percentile values: top-third = high functional, middle-third = middle functional, and bottom-third = low functional lung. For each patient, four treatment plans were created: a combined VQ-guided plan (VQ plan), a ventilation-guided plan (V plan), a perfusion-guided plan (Q plan), and an anatomical plan without functional imaging (A plan). These plans were compared to evaluate differences in dose-volume metrics and the impact of the spatial relationship between the tumor and HFL on radiation dose reduction. A flowchart of the study design is shown in Fig. 1.

**Fig. 1:**
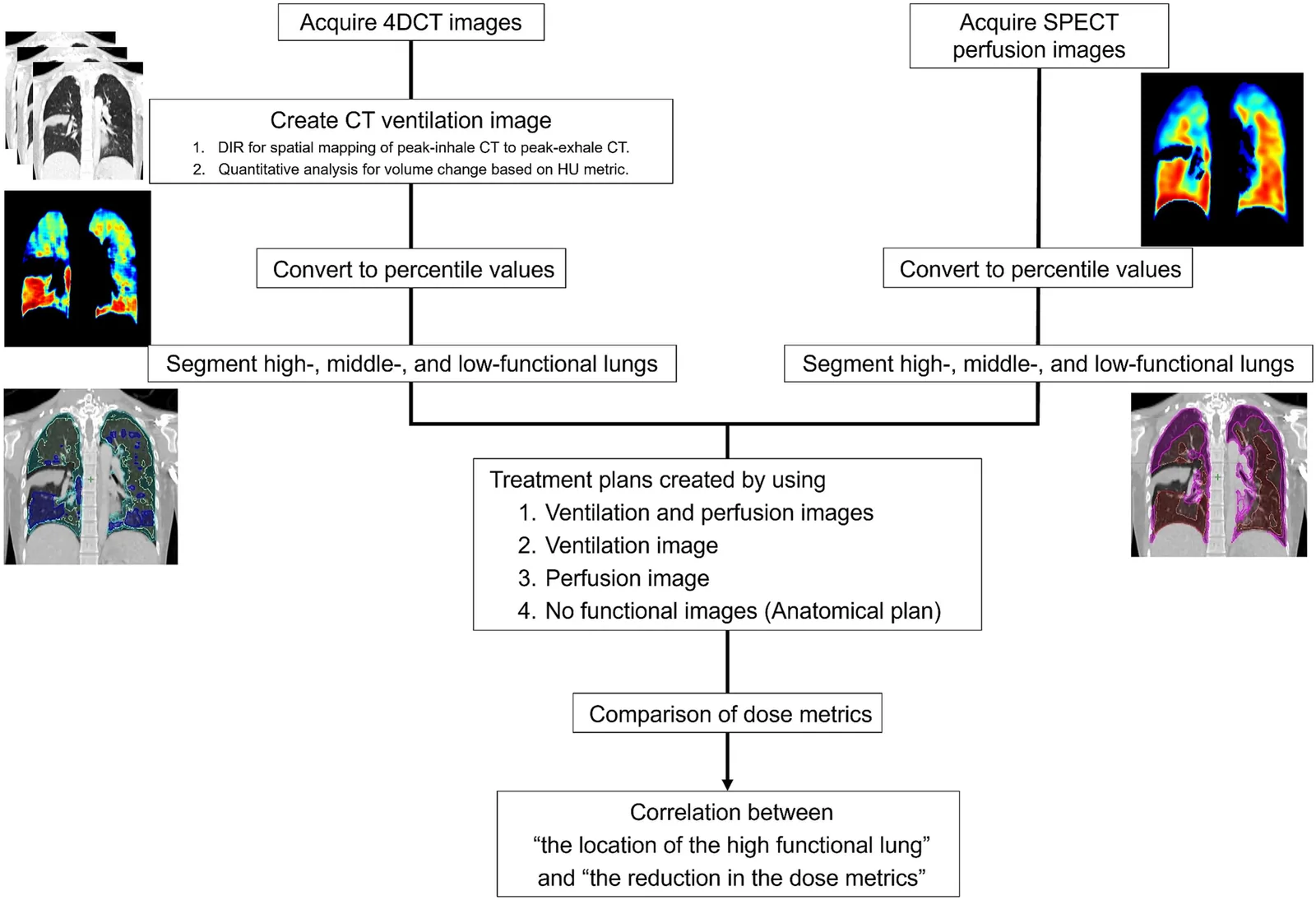
Schematic diagram of the study workflow showing the overall methodology, including the generation of functional lung images, segmentation into functional subregions, treatment planning strategies, and comparative dose-volume metric analysis.

### 2.2 Patients

This retrospective treatment-planning study used existing imaging and planning data from 47 patients with NSCLC prospectively enrolled in a study approved by the institutional review board of Hiroshima University Hospital (No. Rin-206) [11]. The analysis was approved by the Ethics Committee of Komazawa University (No. 20–10), and an opt-out procedure was used in accordance with the Japanese Ethical Guidelines for Medical and Health Research Involving Human Subjects [12]. All data were de-identified before analysis. Patient characteristics are shown in Table 1. All patients underwent both 4DCT and SPECT perfusion scans. Pretreatment scans were acquired with patients in the supine position with arms raised overhead, with a median interval of one day between the two imaging sessions.

**Table 1:** Patient characteristics.

Parameter	Value
Age (years)	66.7 ± 9.6
Sex	
Male	39/47
Female	8/47

Tumor location	
RUL	18/47
RML	4/47
RLL	4/47
LUL	18/47
LLL	3/47

Stage	
I	1/47
II	7/47
III	39/47

PTV volume (cm^3^)	
Overall	373 ± 238
Stage I	124
Stage II	238 ± 108
Stage III	404 ± 246

Patient characteristics are presented for all 47 enrolled patients; subsequent analyses were performed in the 46 patients included in the final analysis.

RUL: right upper lobe, RML: right middle lobe, RLL: right lower lobe, LUL: left upper lobe, LLL: left lower lobe.

### 2.3 Ventilation imaging with 4DCT

Four-dimensional CT images were acquired using a multidetector CT scanner (LightSpeed, GE Medical Systems, Waukesha, WI, USA) in cine mode with a matrix size of 512 × 512 and slice thickness of 2.5 mm, in combination with a real-time position management (RPM) system (Varian Medical Systems, Palo Alto, CA, USA). The acquired data were sorted based on the respiratory phase to reconstruct the 4DCT images. Ventilation images were generated from 4DCT image sets via deformable image registration (DIR) using elastix v4.8, an open-source image registration software package, followed by quantitative image analysis of regional volume changes [13]. These changes, reflected in CT values, served as a surrogate for ventilation. The image acquisition method was the same as that used by Nakajima et al. [14]. Regional volume changes were quantified using Hounsfield unit (HU)-based metrics, as previously described [15], [16], [17]. The DIR parameter settings were the same as those used by Kanai et al. (Parameter Set 2), which validated the geometric accuracy with a target registration error of <1.3 mm [18]. The transformed images and lung ventilation images generated using DIR were visually evaluated by two medical physicists to confirm the absence of clinically significant errors. Following the method described by Vinogradskiy et al., the lung ventilation images were downsampled to match the spatial resolution of the SPECT lung perfusion images and then converted into percentile images [19]. Details are provided in Supplementary Material A.

### 2.4 Perfusion imaging with SPECT

Perfusion imaging with SPECT was performed using a commercial scanner (BrightView XCT, Philips Healthcare, Cleveland, OH, USA) after intravenous administration of 185 MBq of technetium-99 m-labeled macroaggregated albumin (^99m^Tc-MAA). Projections for SPECT imaging were acquired with a 128 × 128 matrix, a pixel size of 3.19 × 3.19 mm^2^, and a slice spacing of 3.19 mm. Detailed procedures for SPECT imaging have been previously described [11]. The perfusion images were converted into percentile distribution images in the same manner as the CT ventilation images.

### 2.5 Anatomical and functional treatment planning

All plans were created using Eclipse v16.1 (Varian Medical Systems, Palo Alto, CA, USA). A total dose of 60 Gy in 30 fractions was prescribed to cover 95% of the planning target volume (PTV). Volumetric-modulated arc therapy (VMAT) was used for all plans, utilizing two full-arc rotations at 6 MV energy, with collimator angles of 300° and 30°. The Acuros XB dose calculation algorithm was employed. Initial optimization parameters were based on prior research (see Table S1 in the Supplementary Material) [7], [10]. Compliance with the dose constraints established in accordance with the Radiation Therapy Oncology Group (RTOG) 0617 protocol was evaluated (see Table S2 in Supplementary Material) [20]. If constraints were not met, optimization parameters, including volume and priority assignments,were iteratively adjusted until compliance was achieved. Cases where constraints could not be satisfied in any of the A, V, Q, or VQ plans were excluded from the analysis (see Fig. S1 in Supplementary Material).

### 2.6 Dose-volume and dose-function metric evaluation

Following previous studies, dose evaluation was performed using region of interest (ROI)-based dose-function metrics: mean lung dose (MLD), V_20 Gy,_ and V_5 Gy_ (the percentage of HFL volume receiving ≥20 Gy or ≥ 5 Gy, respectively) [10]. These metrics were calculated for both HVL and HPL in all treatment plans. In addition, voxel-based dose-function metrics were also assessed. Ventilation-based and perfusion-based dose-function histograms (DFHs) were computed from the corresponding functional images [21]. Three dose-function metrics were calculated: fMLD (MLD weighted by regional function), fV_5 Gy,_ and fV_20 Gy_ (percent lung function receiving 5 and 20 Gy, respectively).

Dose-volume metrics for the whole lung included V_20 Gy_ and the mean dose. Dose-volume metrics for normal organs other than the lungs included the heart V_40 Gy_, the spinal cord maximum dose, and the esophageal mean dose.

The conformity index (CI) was defined according to the method described by Paddick [22]:

(1) $$\mathrm{conformity index}=\frac{{{\mathrm{TV}}_{\mathrm{PTV}}}^{2}}{\left(\mathrm{TV}\times \mathrm{PTV}\right)}\;$$

in which ${\mathrm{TV}}_{\mathrm{PTV}}$ refers to the volume within the PTV greater than or equal to the prescription dose, PTV is the volume of PTV, and TV is the volume surrounded by the prescription dose. The homogeneity index (HI) was determined as follows:

(2) $$\mathrm{homogeneity index}=\frac{D_{5\%}}{D_{95\%}}\;$$

### 2.7 Dose-volume metric correlations with HFL position and PTV volume

To evaluate the impact of tumor location relative to the HFL, the PTV was expanded in 5 mm increments in the anteroposterior and lateral directions. The percentage of overlap with HVL or HPL was calculated using the following equation:

(3) $$\text{Percentage of overlap}=\frac{\text{Overlapped volumes of}\;\mathrm{PTV}\;\text{and}\;\mathrm{HFL}}{\text{volume of}\;\mathrm{HFL}}\times 100\;$$

Spearman's rank correlation coefficient was used to determine the correlation between the percentage of overlap and reduction in MLD, V_20 Gy,_ and V_5 Gy_ for the HVL in the VQ plans compared to the A plans. The same analysis was performed for the HPL. Complementary to the positional analysis, the correlation between PTV volume and dose reduction in each HFL was also assessed by calculating differences in MLD, V_20 Gy_, and V_5 Gy_ between the VQ plans and the A plans. Spearman's rank correlation coefficients were used to evaluate associations between PTV volume and reductions in these dose-volume metrics, analyzed separately for HVL and HPL. All calculations were performed using MATLAB software v9.9 (MathWorks, Natick, MA, USA).

### 2.8 Statistical analysis

Statistical analyses were also performed using MATLAB. The dose-volume and dose-function metrics from each treatment plan were firs*t*-tested for normality using the Shapiro-Wilk test. For normally distributed data, a two-tailed paired *t-*test was performed; for non-normally distributed data, a two-tailed Wilcoxon signed-rank test was applied. This study used a significance level of *p* < 0.008 after Bonferroni correction for comparisons among the four groups: A plans, V plans, Q plans, and VQ plans. Spearman's rank correlation was also employed to determine the correlation between the percentage overlap of the expanded PTV with HFL and the corresponding dose reductions.

## 3 Results

One of 47 patients was excluded because none of the four treatment plans met RTOG dose constraints after priority and volume adjustments. The remaining patients satisfied the dose constraints across all plans. In a representative case showing the greatest HFL dose reduction, visual inspection indicated better HFL preservation with the VQ plan than with the A plan, and the dose-volume histogram (DVH) curves for the HVL and HPL shifted toward lower doses (Fig. 2a, b). Although doses to the PTV and spinal cord were higher in the VQ plan, all values remained within the dose constraints (Fig. 2c).

**Fig. 2:**
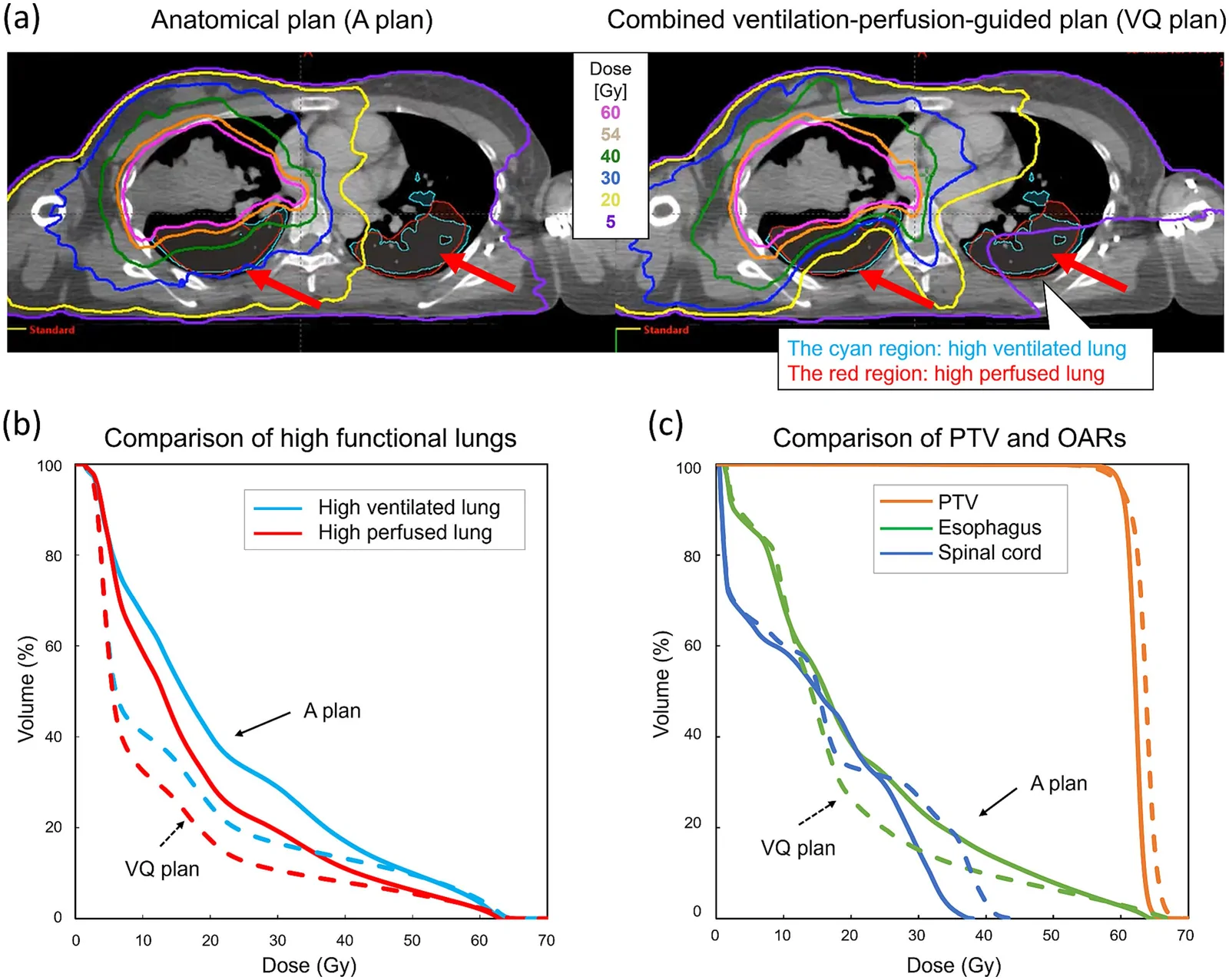
Representative example comparing an anatomical plan and a combined ventilation-perfusion-guided plan in a single patient. (a) Dose distribution maps. (b) DVHs for the HFL. (c) DVHs for the PTV and organs at risk. In (a), solid isodose lines represent dose levels, with colors corresponding to the dose scale; cyan regions indicate the HVL and red regions indicate the HPL. In (b), solid lines represent the A plan and dashed lines represent the VQ plan. The cyan curves correspond to the HVL; the red curves to the HPL. In (c), the PTV is shown in orange, the spinal cord in blue, and the esophagus in green. (For interpretation of the references to colour in this figure legend, the reader is referred to the web version of this article.)

All function-guided plans (V, Q, and VQ) demonstrated significantly lower ROI-based dose-function metrics than the A plans, with the VQ plans consistently showing the lowest values across all metrics (Fig. 3). Compared with the A plans, the VQ plans reduced the MLD from 12.4 to 11.5 Gy in the HVL and from 11.1 to 10.0 Gy in the HPL. In the HVL, the V plans resulted in lower MLD and V_20 Gy_ values than the Q plans (MLD: 11.7 vs. 11.8 Gy, *p* = 0.007; V_20 Gy_: 19.1% vs. 19.9%, *p* < 0.001). In contrast, in the HPL, the Q plans resulted in lower MLD values than the V plans (MLD: 10.2 vs. 10.5 Gy, *p* = 0.005). The dose-function metrics results also showed that the three plans using functional imaging significantly reduced the dose compared to the A plans, with the VQ plans yielding the lowest values (details are shown in Fig. S2 in Supplementary Material).

**Fig. 3:**
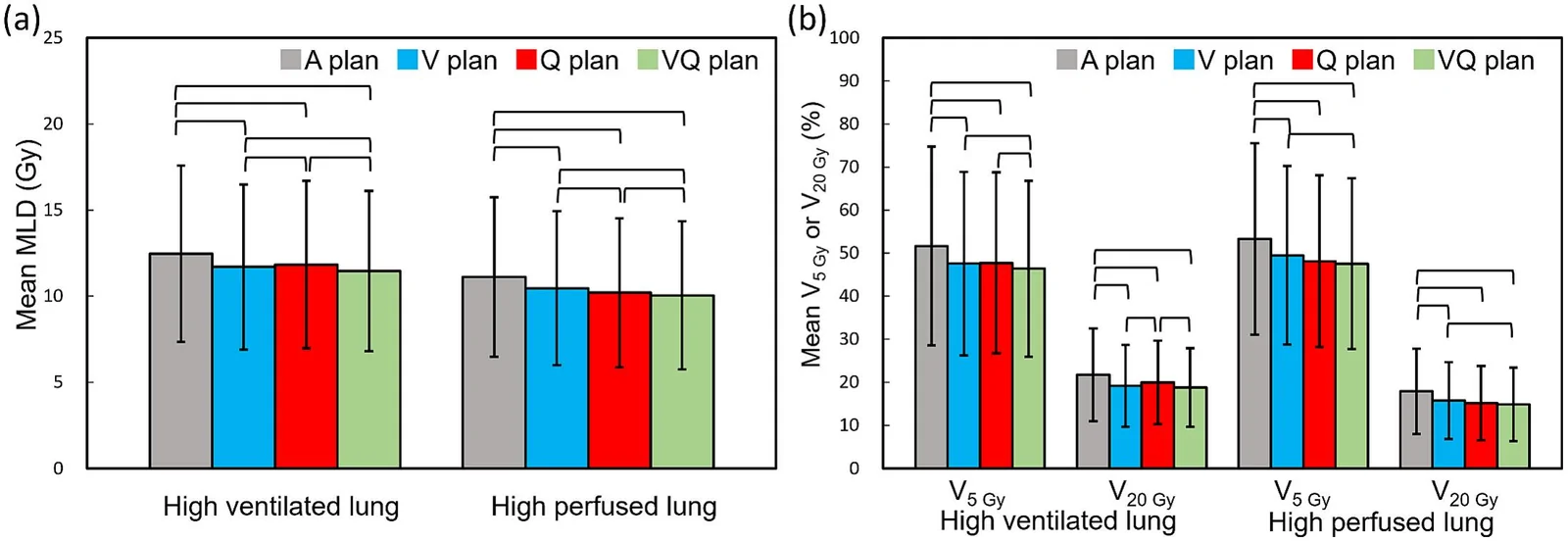
Comparison of ROI-based dose-function metrics among treatment plans in the HFL. (a) MLD. (b) V_5 Gy_ and V_20 Gy_. The A, V, Q, and VQ plans are compared. Bars represent mean values, and error bars indicate standard deviations. Only statistically significant differences are indicated in square brackets.

Compared with the A plans, the average PTV dose increased slightly in the V, Q, and VQ plans. The CI significantly decreased and the HI significantly increased in the function-guided plans. In the whole lung, V_20 Gy_ was significantly reduced in all function-guided plans, whereas the mean dose was significantly reduced only in the Q and VQ plans. Heart V_40 Gy_ was significantly increased in the Q and VQ plans, but not in the V plan, whereas the spinal cord maximum dose was significantly increased in all function-guided plans. No significant differences in the esophageal mean dose were observed among the four plans (Table 2).

**Table 2:** Dose assessment in PTV and organs at risk.

	Anatomical plan	Functional plans		
	A plan	V plan	Q plan	VQ plan
PTV; Mean dose (Gy)	62.8 ± 0.8	63.2 ± 0.7 (*p* < 0.001)	63.2 ± 0.8 (p < 0.001)	63.4 ± 0.7 (p < 0.001)
PTV; CI	0.88 ± 0.06	0.86 ± 0.08 (p < 0.001)	0.86 ± 0.08 (p < 0.001)	0.85 ± 0.08 (p < 0.001)
PTV; HI	1.08 ± 0.02	1.09 ± 0.02 (p < 0.001)	1.09 ± 0.02 (p < 0.001)	1.10 ± 0.02 (p < 0.001)
Whole lung; V_20 Gy_ (%)	22.9 ± 8.6	22.1 ± 8.7 (*p* < 0.001)	22.0 ± 8.4 (*p* = 0.001)	21.5 ± 8.3 (p < 0.001)
Whole lung; Mean dose (Gy)	12.8 ± 4.0	12.7 ± 4.2 (*p* = 0.026)	12.6 ± 4.1 (*p* = 0.006)	12.4 ± 4.0 (*p* < 0.001)
Heart; V_40 Gy_ (%)	2.8 ± 4.3	3.4 ± 5.4 (*p* = 0.010)	3.5 ± 5.6 (*p* < 0.001)	3.6 ± 6.1 (*p* = 0.001)
Spinal cord; Maximum dose (Gy)	39.7 ± 8.0	41.4 ± 7.1 (p < 0.001)	41.8 ± 7.4 (p < 0.001)	42.4 ± 6.8 (p < 0.001)
Esophagus; Mean dose (Gy)	21.5 ± 8.1	21.5 ± 8.3 (*p* = 0.467)	21.6 ± 8.4 (*p* = 0.489)	21.5 ± 8.4 (*p* = 0.794)

Abbreviations: CI, Conformity index; HI, Homogeneity index; PTV, planning target volume; A plan, anatomical plan; V plan, ventilation-guided plan; Q plan, perfusion-guided plan; VQ plan, combined ventilation-perfusion-guided plan.

Data are presented as mean ± standard deviation. Statistical significance was assessed by comparing each functional plan to the anatomical plan, with corresponding *p*-values recorded.

In the HVL, overlap correlated with reductions in MLD (ρ = 0.50, Spearman's rank correlation coefficient) and V_20 Gy_ (ρ = 0.66), with the strongest correlations at a 25 mm PTV expansion, while V_5 Gy_ peaked at ρ = 0.40 with a 50 mm expansion. For HPL, the correlation with MLD was strongest at 45 mm (ρ = 0.31), whereas the strongest correlations with V_20 Gy_ and V_5 Gy_ were observed at 35 mm (ρ = 0.44) and 50 mm (ρ = 0.35), respectively (Fig. 4). Correlations between voxel-based dose-function metrics reductions and overlap with the expanded PTV are shown in Fig. S3 in Supplementary Material: fMLD and fV_20 Gy_ showed stronger correlations when HFL were closer to the PTV, whereas fV_5 Gy_ correlated more strongly when the tumor was farther away. Correlations between PTV volume and dose reduction were weak across all metrics (maximum absolute ρ = 0.20; Fig. S4 in Supplementary Material).

**Fig. 4:**
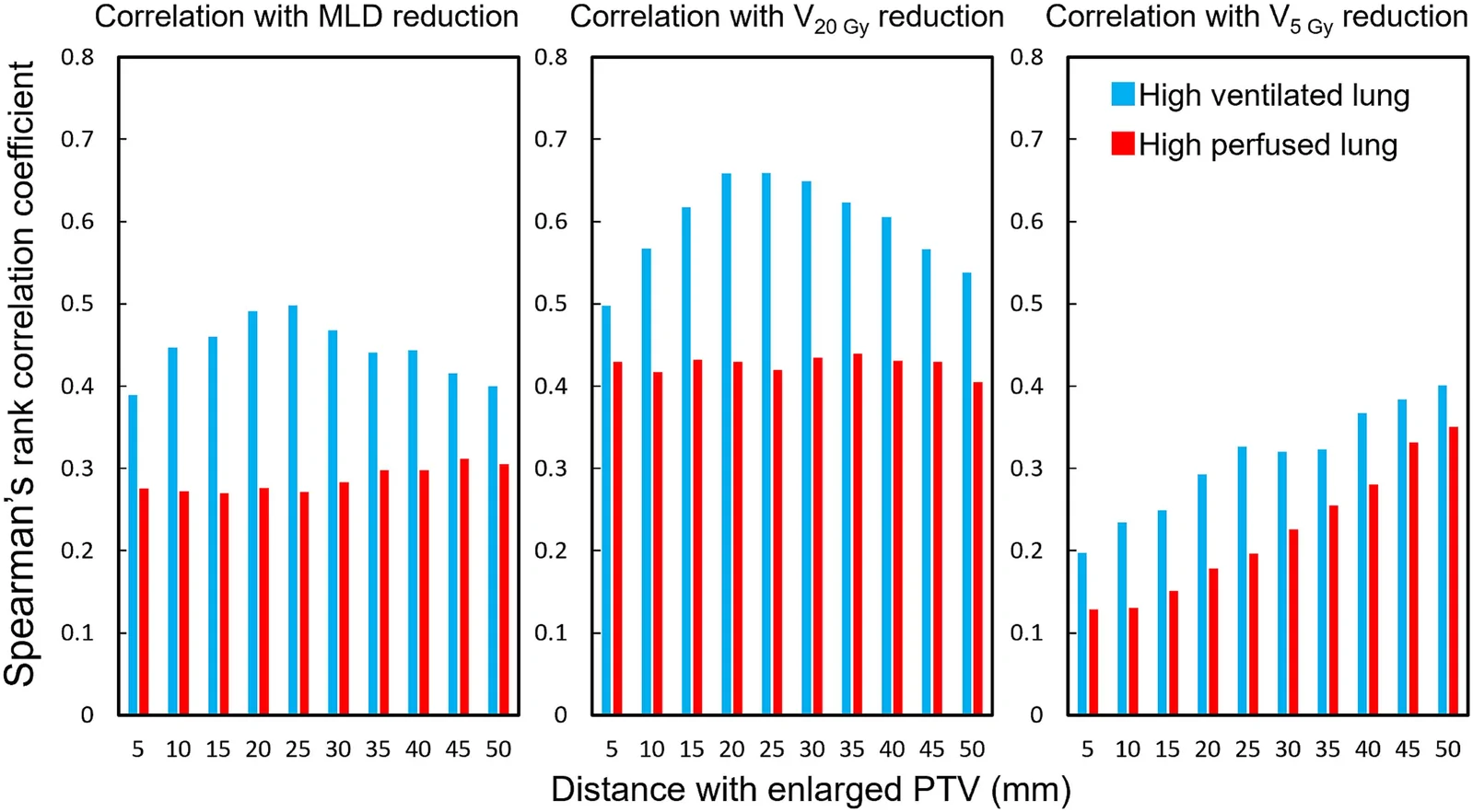
Correlation between dose reduction in ROI-based dose-function metrics and overlap with expanded PTV. Dose reductions were calculated as the difference between the VQ and A plans, and were correlated with the overlap ratio between the expanded PTV and the HFL. Cyan: HVL; red: HPL. (For interpretation of the references to colour in this figure legend, the reader is referred to the web version of this article.)

## 4 Discussion

This study demonstrated that incorporating lung functional imaging into radiotherapy planning significantly reduced the radiation dose to HFL compared with anatomical planning alone. Furthermore, the combined use of ventilation and perfusion imaging improved HFL sparing compared with planning based on either functional imaging modality alone. Functional lung avoidance planning based on a single functional imaging modality may be insufficient to adequately reduce the dose to HFL identified by the other modality. In addition, regions within 25–45 mm of the PTV were most strongly associated with reductions in MLD and V_20 Gy_.

The present findings were consistent with previous reports on function-guided radiotherapy [8], [10], [23]. Regarding combined functional planning, Bucknell et al. [8] utilized ^68^Ga-PET/CT and reported an MLD reduction of approximately 1.9 Gy and a 4.7% decrease in fV_20 Gy_ within HFL compared with anatomical plans. Despite differences in imaging modalities, these reported benefits are consistent with the present results. Furthermore, the present findings aligned with earlier studies utilizing identical imaging modalities; Yamamoto et al. [10] showed a 2.0 Gy MLD reduction to HVL using DIR-based images, and Shioyama et al. [23] achieved a 4.2 Gy MLD reduction to HPL using ^99m^Tc-MAA SPECT.

Using normal tissue complication probability (NTCP) modeling, Faught et al. reported a 7.1% reduction in the predicted risk of grade ≥ 2 RP with 4DCT ventilation avoidance planning [24]. Although ventilation-guided planning can reduce lung toxicity, the findings of this study suggest that ventilation alone may not sufficiently spare HPL; integrating ventilation and perfusion may better preserve HFL and further reduce RP risk. Regarding cardiac dose, previous literature has reported that higher heart V_40 Gy_ is associated with poorer overall survival [25]. At the cohort level in the present study, heart V_40 Gy_ increased modestly from 2.8% in the A plans to 3.4–3.6% in the function-guided plans. Regarding the spinal cord, QUANTEC guidelines estimate the risk of radiation myelopathy at 54 Gy to be <1% [26], and all cases strictly satisfied the predefined constraint of ≤50.5 Gy. Therefore, the overall clinical impact is considered limited. However, in some patients, functional lung sparing must be carefully balanced against protection of the heart and spinal cord during individualized treatment planning. Unlike previous combined ventilation-perfusion avoidance studies, which predominantly used PET-based imaging, this study combined 4DCT ventilation with SPECT perfusion, supporting the planning feasibility of this modality combination.

Regarding the impact of tumor location, Yamamoto et al. demonstrated that dose reduction was greater when the PTV was closer to HVL in functional avoidance planning incorporating 4DCT ventilation imaging, using a binary classification based on the extent of overlap between the PTV and HVL [10]. Building on their findings, the present study used 5 mm incremental PTV expansions to provide a more detailed characterization of this spatial relationship. Regions of HFL located within 25–45 mm of the PTV contributed most significantly to reductions in MLD and V_20 Gy_. In functional lung avoidance planning, the volume of HFL within 25–45 mm of the PTV may therefore serve as a useful indicator for identifying patients likely to benefit from dose reduction. Furthermore, selectively applying stronger optimization constraints to HFL within this range may enhance dose reduction. In contrast, PTV volume showed only weak correlations with dose reduction (maximum absolute ρ = 0.20), suggesting that PTV-HFL proximity is a more important determinant of dose-sparing than target volume alone.

One of the challenges for clinical implementation is the acquisition of perfusion imaging. The use of 4DCT is increasingly common in the treatment of lung cancer, making ventilation imaging readily available without additional scans. On the other hand, perfusion imaging still requires additional examinations such as PET or SPECT. Therefore, the development of more efficient and accessible methods for perfusion imaging is warranted.

This study has three limitations. First, uneven tumor distribution precluded robust subgroup or lobe-specific analyses; upper-lobe tumors accounted for 78.3% (36/46) of the analyzed cases, with relatively few tumors in the middle and lower lobes. Larger, balanced cohorts are needed to determine whether the benefits of ventilation-perfusion-based functional lung avoidance planning vary by tumor location. Second, differences between ventilation and perfusion imaging modalities may introduce variability in spatial resolution and physiological assumptions, potentially affecting functional assessment. Third, comprehensive NTCP models integrating ventilation and perfusion remain limited. Further studies, including prospective clinical trials, are needed to validate the clinical effectiveness of this approach and establish standardized dose-volume constraints.

In conclusion, incorporating lung functional imaging into radiotherapy planning significantly reduced dose to HFL compared with anatomical approaches. Greater dose reduction was achieved when both ventilation and perfusion information was integrated than when either was used alone, suggesting that combined functional imaging provides a more effective strategy for functional lung avoidance.

## CRediT authorship contribution statement

**Genta Michimata:** Writing – review & editing, Writing – original draft, Validation, Investigation, Conceptualization. **Yujiro Nakajima:** Writing – review & editing, Validation, Conceptualization. **Tomoki Kimura:** Writing – review & editing, Validation, Conceptualization. **Nobuki Imano:** Writing – review & editing, Validation, Conceptualization. **Shinya Ito:** Writing – review & editing, Validation, Conceptualization. **Yukio Fujita:** Writing – review & editing, Validation, Conceptualization. **Naoki Tohyama:** Writing – review & editing, Validation, Conceptualization.

## Ethics declaration

The authors have NOT obtained informed consent from participants or their legal representatives. This study was a retrospective planning study that used only existing clinical imaging and treatment planning data; no new samples or information were collected from the participants. In accordance with the Japanese Ethical Guidelines for Medical and Health Research Involving Human Subjects, which permit the use of an opt-out procedure in place of individual written or verbal informed consent for retrospective studies using existing information, the requirement for informed consent was waived by the institutional review board of Hiroshima University Hospital, which approved the study (reference No. Rin-206). Information about the study was made publicly available on the institutional website of Hiroshima University, and participants were given the opportunity to decline participation at any time. Data from any participant who declined would have been removed from the analysis and destroyed immediately. All data were de-identified before analysis, and no individually identifiable information is presented in this article.

This study was performed in compliance with relevant laws, regulatory frameworks and guidelines where the research took place. This study was conducted in accordance with Japanese Ethical Guidelines for Medical and Health Research Involving Human Subjects. This study was approved by the Hiroshima University Hospital (Approval No. Rin-206) and Komazawa University (Approval No. 20-10).

## Declaration of generative AI and AI-assisted technologies in the writing process

During the preparation of this work the authors used ChatGPT (OpenAI) and DeepL (DeepL SE) in order to improve language and readability. After using these tools/services, the authors reviewed and edited the content as needed and take full responsibility for the content of the publication.

## Funding

This work was supported by 10.13039/501100001691JSPS KAKENHI Grant Number 25K10921.

## Declaration of competing interest

The authors declare that they have no known competing financial interests or personal relationships that could have appeared to influence the work reported in this paper.

## Data Availability

Raw CT images are not publicly available due to institutional privacy restrictions; however, de-identified patient-level dose-volume and dose-function data are provided in the Supplementary Material (Tables S3–S5).

## Appendix A Supplementary data

Supplementary Material: Detailed methods for four-dimensional computed tomography-based ventilation imaging, treatment planning optimization parameters and dose constraints, the treatment planning workflow, additional dose-function analyses, and patient-level dose-volume and dose-function metrics (Tables S1–S5 and Figs. S1–S4).
