# Supplementary material for: Functional lung avoidance planning with combined ventilation-perfusion imaging versus individual functional imaging in lung cancer radiotherapy
Source: Phys Imaging Radiat Oncol. 2026 Nov 3;42:101118. doi: 10.1016/j.phro.2026.101118 (PMC13760710; doi:10.1016/j.phro.2026.101118)
Supplement: Supplementary file 1 [file mmc1.pdf]

Supplementary material A

Ventilation imaging based on computed tomography

The four-dimensional computed tomography (4DCT) data and CT ventilation images used in the present study were obtained from the same patient cohort and generated using the same methodology as those reported previously by Nakajima et al. [1]. The 4DCT scans were acquired using a multidetector CT scanner (LightSpeed, GE Medical Systems, Waukesha, WI, USA) in cine mode in combination with a real-time position management system (Varian Medical Systems, Palo Alto, CA, USA). The real-time position management system provided a surrogate respiratory signal based on temporal abdominal wall motion, which was used together with the cine-mode CT images to sort the data into ten respiratory-phase CT images using an image analysis workstation (AdvantageSim, GE Healthcare, Princeton, NJ, USA). The acquisition parameters were as follows: tube voltage, 120 kVp; tube current, 80 mA; scan time, 0.5 s; slice thickness, 2.5 mm; and matrix size, 512 × 512.

Ventilation images were generated from the 4DCT image sets using deformable image registration (DIR), and regional volume changes were quantified as a surrogate for ventilation. Using nonparametric volume-based DIR, the peak-inhalation 4DCT image (moving) was spatially mapped to the peak-exhalation image (fixed), yielding a displacement vector field using elastix v4.8, an open-source software, for image registration [2]. The DIR parameter settings were the same as the Parameter Set 2 reported in a study by Kanai et al. which validated the geometric accuracy with a target registration error of < 1.3 mm [3]. Regional volume changes were quantified using Hounsfield unit (HU)-based metric [4-6]. The resulting HU-based ventilation metric (V_{HU}) was defined by the following formula:

$$V_{HU}(x,y,z) = \frac{HU_{ex}(x,y,z) - HU_{in}\left\{x+u_x(x,y,z), y+u_y(x,y,z), z+u_z(x,y,z)\right\}}{\left[HU_{in}\left\{x+u_x(x,y,z), y+u_y(x,y,z), z+u_z(x,y,z)\right\}+1000\right]}\rho_{scaling}$$

where HU is the HU value and u(x, y, z) is a displacement vector mapping the voxel at location (x, y, z) of the peak exhalation image (ex) to the corresponding location of the peak-inhalation image (in). $r_{scaling}$ is the CT density scaling factor; $r_{scaling} = (HU_{ex} + 1024)/774$, which is a value ranging from 0 for the voxel with the lowest lung CT density (-1024 HU) to 1 or the voxel with the highest density (-250 HU) to transform a purely mechanical model of regional ventilation based on volume change alone to a more physiologic model, in a manner similar to that described by Hegi Johnson et al. [7]. As described by Guerrero et al. mass correction was applied to HUin to account for the difference in lung mass between peak-exhalation and peak-inhalation phases [5]. To reduce the influence of CT noise, a median filter with a kernel width of $7 \times 7 \times 7$ voxels^3 was applied before calculating V_{HU}, as described by Hegi-Johnson et al. [7]. The ventilation images and deformation maps were visually inspected to check for any major errors. The CT ventilation images were down-sampled to match the spatial resolution of the SPECT perfusion images and converted into percentile distribution images in a manner similar to that described by Vinogradskiy et al. [8].

[1] Nakajima Y, Kadoya N, Kimura T, Hioki K, Jingu K, Yamamoto T. Variations between dose-ventilation and dose-perfusion metrics in radiation therapy planning for lung cancer. Adv Radiat Oncol 2020;5:459-65. https://doi.org/10.1016/j.adro.2020.03.002.

[2] Klein S, Staring M, Murphy K, Viergever MA, Pluim JPW. elastix: a toolbox for intensity-based medical image registration. IEEE Trans Med Imaging 2010;29:196-205. https://doi.org/10.1109/TMI.2009.2035616.

[3] Kanai T, Kadoya N, Ito K, Onozato Y, Cho SY, Kishi K, et al. Evaluation of accuracy of B-spline transformation-based deformable image registration with different parameter settings for thoracic images. J Radiat Res 2014;55:1163-70. https://doi.org/10.1093/jrr/rru062.

[4] Guerrero T, Sanders K, Noyola-Martinez J, Castillo E, Zhang Y, Tapia R, et al. Quantification of regional ventilation from treatment planning CT. Int J Radiat Oncol Biol Phys 2005;62:630-4. https://doi.org/10.1016/j.ijrobp.2005.03.023.

[5] Guerrero T, Sanders K, Castillo E, Zhang Y, Bidaut L, Pan T, et al. Dynamic ventilation imaging from four-dimensional computed tomography. Phys Med Biol 2006;51:777-91. https://doi.org/10.1088/0031-9155/51/4/002.

[6] Fuld MK, Easley RB, Saba OI, Chon D, Reinhardt JM, Hoffman EA, et al. CT-measured regional specific volume change reflects regional ventilation in supine sheep. J Appl Physiol 2008;104:1177-84. https://doi.org/10.1152/japplphysiol.00212.2007.

[7] Hegi-Johnson F, Keall P, Barber J, Bui C, Kipritidis J. Evaluating the accuracy of 4D-CT ventilation imaging: First comparison with Technegas SPECT ventilation. Med Phys 2017;44:4045-55. https://doi.org/10.1002/mp.12317.

[8] Vinogradskiy Y, Castillo R, Castillo E, Tucker SL, Liao Z, Guerrero T, et al. Use of 4-dimensional computed tomography-based ventilation imaging to correlate lung dose and function with clinical outcomes. Int J Radiat Oncol Biol Phys 2013;86:366-71. https://doi.org/10.1016/j.ijrobp.2013.01.004.

Table S1. Initial value of optimization parameters for anatomical and functional plans

			Anatomical plan			Functional plan		
			Volume (%)	Dose (cGy)	Priority	Volume (%)	Dose (cGy)	Priority
PTV		upper	0	6300	70	0	6300	70
		upper	10	6171	70	10	6171	70
		lower	100	5700	70	100	5700	70
		lower	98	6000	80	98	6000	80
Body		upper	0	6428	50	0	6428	50
Spinal Cord		upper	0	4500	90	0	4500	90
Esophagus		upper	0	6428	50	0	6428	50
		upper	35	5500	50	35	5500	50
Heart		upper	50	4500	50	50	4500	50
Lung		upper	15	2000	70	--	--	--
		upper	40	500	70	--	--	--
Functional Lung	High	upper	--	--	--	15	2000	130
		upper	--	--	--	40	500	130
	Middle	upper	--	--	--	25	2000	100
	Low	upper	--	--	--	25	2000	70

Abbreviations: PTV, planning target volume.

Table S2. Dose constraints in this study based on the RTOG 0617 protocol

	Dose constraints
PTV	$D_{95\%}$ = 60 Gy
	D_{max} < 72 Gy (Acceptable: < 75 Gy)
Spinal Cord	D_{max} < 50.5 Gy
Lung	$V_{20\ Gy}$ < 37%
Esophagus	D_{mean} < 34 Gy
Heart	$V_{60\ Gy}$ < 33.3%
	$V_{45\ Gy}$ < 66.7%
	$V_{40\ Gy}$ < 100%

Abbreviations: PTV, planning target volume.

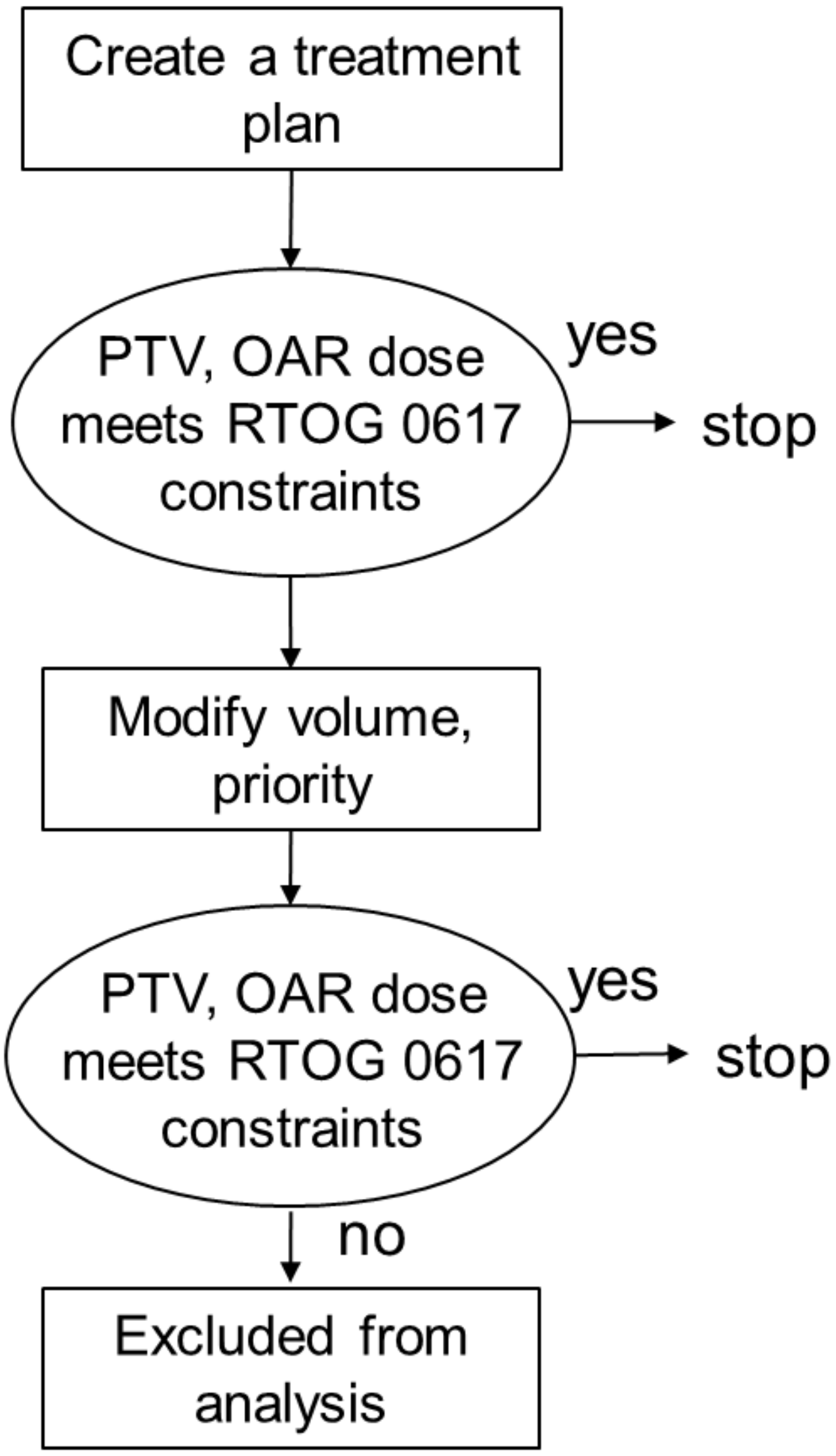

Fig. S1. Schematic diagram showing the creation of clinically acceptable treatment plans. Optimization parameters, including volume objectives and priority weights, were iteratively adjusted to satisfy the RTOG 0617 dose constraints.

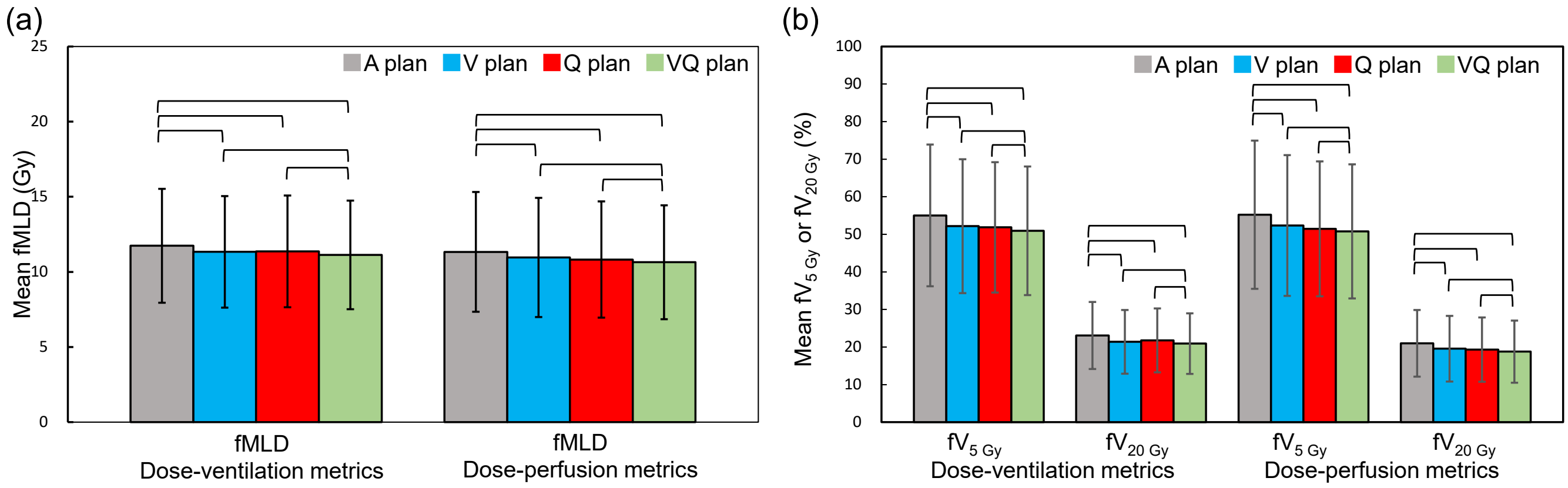

Fig. S2. Comparison of voxel-based dose–function metrics among the A, V, Q, and VQ plans: (a) fMLD and (b) $fV_{5\,Gy}$ and $fV_{20\,Gy}$. Bars represent mean values, and error bars indicate standard deviations. Only statistically significant differences are indicated in square brackets.

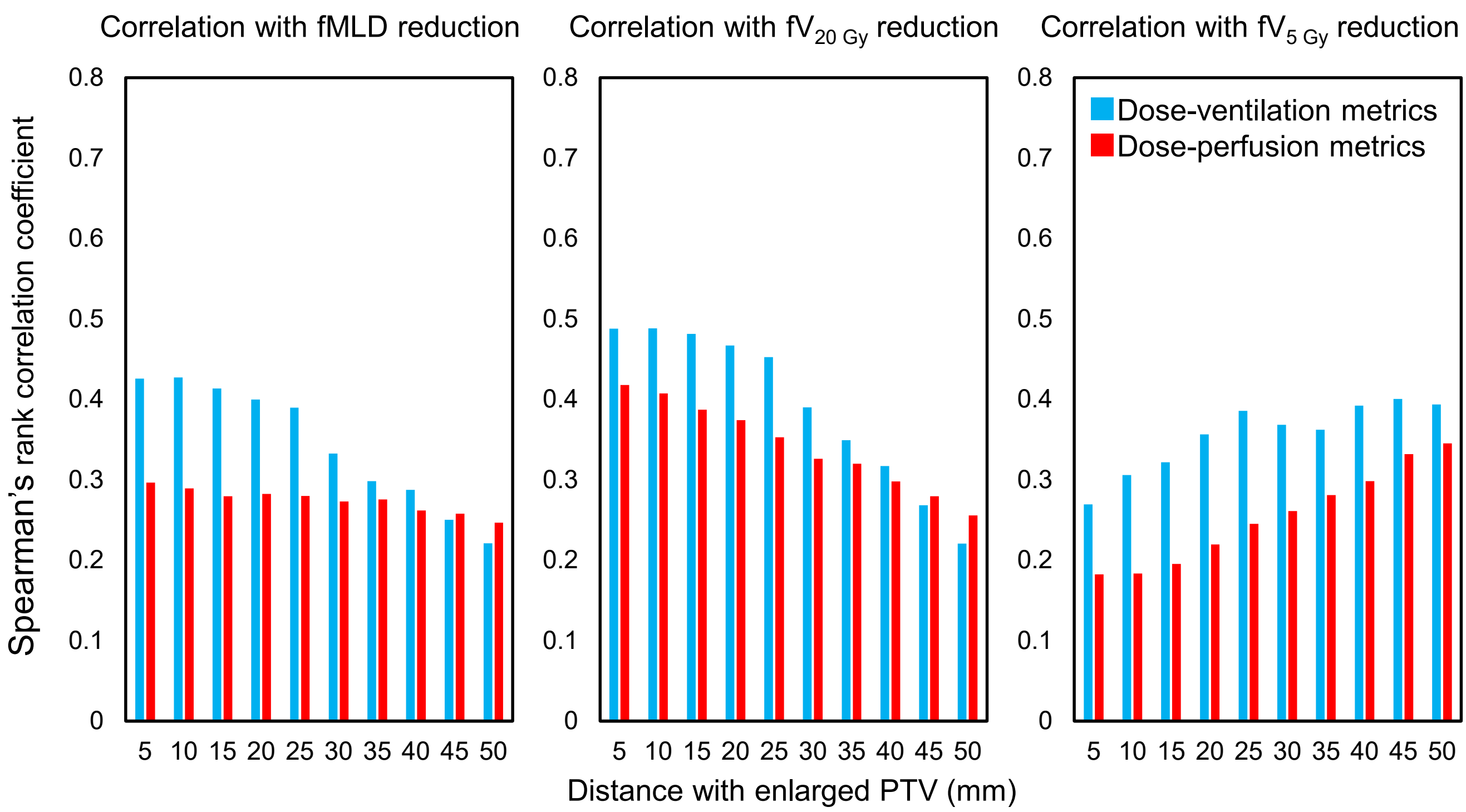

Fig. S3. Correlation between dose reduction in voxel-based dose-function metrics and overlap with extended PTV. Correlation between dose reduction in the combined VQ plans relative to the A plans and the overlap ratio between the expanded PTV and the high functional lungs. Blue: correlation for the high ventilated lung; Red: correlation for the high perfused lung.

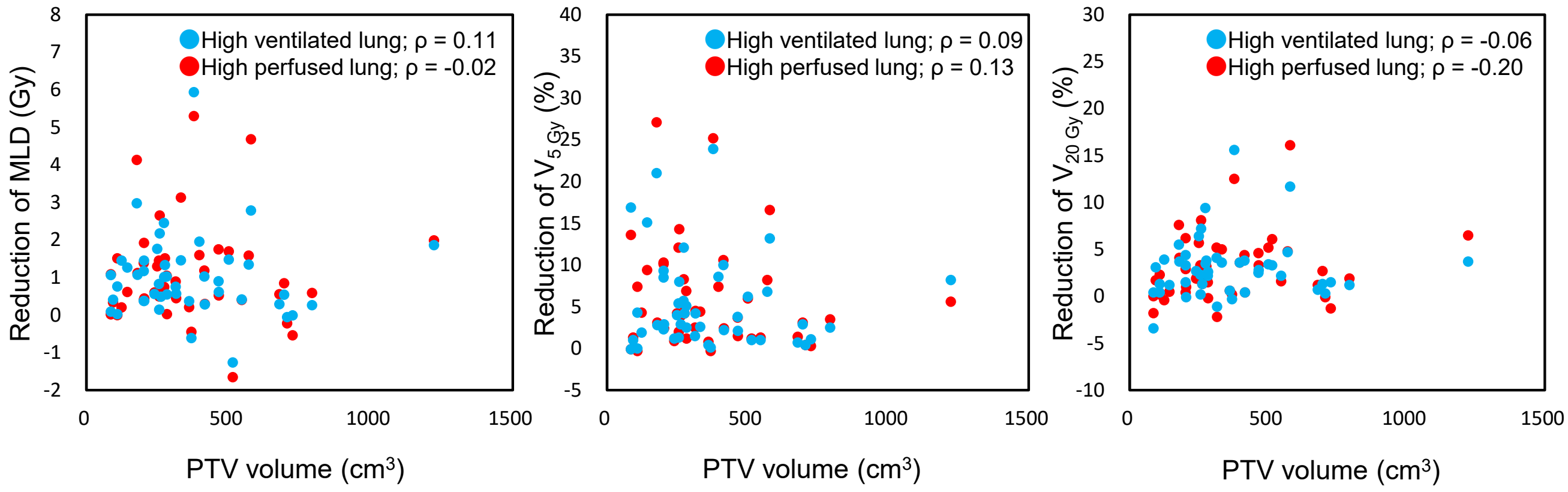

Fig. S4. Spearman's rank correlations between PTV volume and dose reduction in ROI-based dose-function metrics. Dose reductions were calculated by subtracting the values in the VQ plans from those in the A plans. Blue and red indicate data for the high ventilated and high perfused lungs, respectively.

Table S3. Patient-level dose–volume metrics for the planning target volume and organs at risk across the four treatment plans.

Patient ID	PTV volume (cm^3)	PTV: Dmean (Gy)				PTV: CI				PTV: HI			
		A plan	V plan	Q plan	VQ plan	A plan	V plan	Q plan	VQ plan	A plan	V plan	Q plan	VQ plan
1	249.2	62.5	63.8	63.7	64.1	0.91	0.89	0.88	0.88	1.07	1.11	1.10	1.11
2	255.3	62.6	63.9	62.8	64.2	0.93	0.92	0.93	0.92	1.08	1.11	1.08	1.11
3	416.3	62.9	63.0	63.4	63.4	0.79	0.79	0.79	0.79	1.09	1.09	1.07	1.10
4	706.4	63.1	63.5	63.6	63.5	0.83	0.81	0.81	0.81	1.09	1.10	1.10	1.10
5	179.3	64.2	62.0	62.3	62.1	0.65	0.76	0.75	0.73	1.12	1.07	1.08	1.07
6	678.8	62.5	62.5	63.6	63.5	0.94	0.94	0.92	0.92	1.08	1.08	1.10	1.10
7	546.7	62.9	63.4	62.8	63.3	0.93	0.92	0.93	0.92	1.08	1.10	1.08	1.10
8	203.1	62.3	62.7	62.8	62.8	0.90	0.89	0.89	0.89	1.08	1.09	1.09	1.09
9	696.0	62.8	62.7	63.1	63.0	0.91	0.91	0.90	0.91	1.09	1.08	1.09	1.09
10	272.6	62.9	63.1	63.6	63.4	0.86	0.73	0.70	0.74	1.08	1.10	1.11	1.10
11	466.1	62.6	63.6	63.4	63.8	0.83	0.81	0.81	0.81	1.08	1.10	1.10	1.11
12	332.0	63.5	63.7	64.4	64.5	0.88	0.80	0.77	0.78	1.11	1.11	1.12	1.13
13	93.8	61.8	62.3	62.1	62.5	0.92	0.92	0.92	0.92	1.06	1.07	1.07	1.07
14	276.3	62.2	62.6	63.0	63.0	0.92	0.92	0.90	0.91	1.06	1.08	1.09	1.09
15	315.3	62.8	62.8	63.3	63.4	0.91	0.91	0.91	0.90	1.08	1.08	1.09	1.10
16	108.5	61.9	62.8	63.9	64.3	0.90	0.90	0.87	0.86	1.06	1.08	1.11	1.12
17	201.6	62.4	64.5	62.4	62.7	0.91	0.92	0.92	0.92	1.07	1.08	1.07	1.08
18	725.8	63.2	63.3	62.8	63.4	0.71	0.69	0.74	0.71	1.10	1.10	1.09	1.10
19	361.1	62.3	63.4	62.4	63.9	0.93	0.91	0.93	0.89	1.07	1.10	1.07	1.11
20	313.8	62.5	63.3	63.2	63.4	0.90	0.88	0.88	0.88	1.07	1.10	1.09	1.10
21	514.7	63.1	62.9	62.5	62.6	0.83	0.66	0.67	0.66	1.09	1.08	1.08	1.08
22	273.7	65.7	65.7	66.0	64.5	0.82	0.81	0.79	0.82	1.16	1.16	1.17	1.12
23	256.0	62.4	63.8	63.7	64.1	0.93	0.92	0.92	0.91	1.07	1.10	1.10	1.11
24	282.0	63.6	62.1	61.9	62.8	0.87	0.83	0.82	0.85	1.10	1.06	1.06	1.08
25	85.6	62.5	62.7	63.3	63.8	0.91	0.90	0.87	0.87	1.08	1.08	1.09	1.11
26	262.5	63.3	63.8	63.8	64.0	0.89	0.88	0.88	0.88	1.09	1.11	1.11	1.11
27	239.6	62.3	63.5	63.2	63.5	0.91	0.89	0.90	0.89	1.07	1.10	1.09	1.09
28	257.6	62.4	63.4	63.7	64.0	0.87	0.85	0.82	0.82	1.07	1.09	1.10	1.11
29	368.9	63.7	63.1	63.6	63.5	0.70	0.55	0.55	0.52	1.13	1.11	1.13	1.12
30	414.6	64.8	63.9	63.7	64.0	0.82	0.79	0.77	0.78	1.14	1.12	1.12	1.12
31	1222.6	63.4	64.1	64.5	64.5	0.94	0.93	0.92	0.92	1.10	1.12	1.11	1.12
32	465.0	63.2	64.3	64.6	65.0	0.91	0.87	0.86	0.85	1.09	1.12	1.12	1.13
33	85.6	63.2	63.8	63.7	63.4	0.84	0.83	0.83	0.82	1.09	1.11	1.10	1.11
34	793.9	62.4	62.9	62.9	63.0	0.93	0.93	0.93	0.93	1.07	1.09	1.09	1.09
35	283.0	63.3	64.2	63.5	63.6	0.90	0.89	0.91	0.90	1.09	1.11	1.10	1.10
36	377.7	62.1	62.8	62.8	63.5	0.92	0.89	0.89	0.86	1.07	1.09	1.08	1.10
37	201.4	62.8	63.0	62.8	63.0	0.85	0.84	0.84	0.83	1.08	1.09	1.08	1.09
38	396.9	62.4	62.7	62.5	62.8	0.90	0.88	0.89	0.88	1.07	1.08	1.07	1.09
39	108.4	61.6	62.2	62.1	61.9	0.91	0.90	0.90	0.92	1.06	1.06	1.06	1.06
40	123.9	62.6	63.3	63.2	64.3	0.91	0.89	0.89	0.88	1.07	1.08	1.08	1.11
41	177.2	62.2	62.6	62.6	62.7	0.91	0.90	0.90	0.90	1.07	1.08	1.08	1.08
42	579.3	63.2	63.8	63.7	64.3	0.94	0.90	0.90	0.88	1.09	1.10	1.10	1.12
43	143.6	63.3	63.1	63.4	62.0	0.87	0.87	0.85	0.90	1.09	1.09	1.09	1.06
44	570.6	62.3	62.3	62.5	62.7	0.93	0.93	0.93	0.93	1.07	1.07	1.07	1.08
45	501.1	62.3	62.9	62.7	62.9	0.93	0.91	0.92	0.91	1.07	1.08	1.08	1.08
46	202.1	62.3	62.4	62.6	62.8	0.92	0.92	0.91	0.92	1.07	1.07	1.07	1.08

Abbreviations: A plan, anatomical plan; V plan, ventilation-guided plan; Q plan, perfusion-guided plan; VQ plan, combined ventilation–perfusion-guided plan; PTV, planning target volume; CI, conformity index; HI, homogeneity index.

Table S3 (continued). Patient-level dose–volume metrics for the planning target volume and organs at risk across the four treatment plans.

Patient ID	Spinal Cord: Dmax (Gy)				Esophagus: Dmean (Gy)				Heart: $V_{40\ Gy}$ (%)				Lung: $V_{20\ Gy}$ (%)				Lung: Dmean (Gy)			
	A plan	V plan	Q plan	VQ plan	A plan	V plan	Q plan	VQ plan	A plan	V plan	Q plan	VQ plan	A plan	V plan	Q plan	VQ plan	A plan	V plan	Q plan	VQ plan
1	33.8	37.5	38.1	42.1	21.4	21.2	21.4	20.9	6.8	8.4	7.7	9.0	26.7	27.2	26.8	26.9	14.3	14.5	14.2	14.2
2	48.1	50.4	48.8	50.4	7.3	6.1	5.8	5.9	0.0	0.0	0.0	0.0	13.3	12.5	11.8	12.7	9.8	9.5	9.6	9.4
3	41.7	41.6	42.0	41.5	17.5	17.4	17.3	17.2	2.0	1.9	1.8	1.9	27.2	27.5	26.6	26.6	16.4	16.3	16.1	16.1
4	50.2	49.8	50.2	50.3	33.7	33.6	33.8	33.9	0.0	0.0	0.0	0.0	31.6	31.7	30.6	31.8	13.3	13.5	13.3	13.5
5	49.4	43.5	43.3	40.8	30.6	28.7	29.6	28.8	1.1	0.4	0.4	0.7	34.7	34.1	32.4	35.8	18.5	18.2	17.9	18.6
6	46.6	46.4	47.5	47.2	22.6	22.7	23.0	22.9	0.0	0.0	0.0	0.0	20.2	18.1	17.8	17.8	9.9	9.5	9.3	9.3
7	49.2	50.0	49.3	49.9	32.7	32.5	33.0	32.6	5.2	4.8	5.5	4.8	23.3	22.2	22.8	22.2	12.1	11.8	12.1	11.8
8	32.4	33.8	34.1	33.8	16.0	15.8	15.6	15.6	5.3	5.5	5.5	5.5	17.7	17.6	17.4	17.4	9.2	9.1	9.0	9.0
9	48.9	48.6	48.5	48.5	33.9	33.9	34.0	33.8	2.2	2.4	2.7	2.5	20.1	21.5	20.6	20.6	13.0	12.5	12.2	12.3
10	35.4	44.7	48.8	49.3	32.1	33.5	33.6	32.5	1.3	10.1	14.3	9.9	31.5	32.6	33.4	28.7	16.0	16.6	16.7	15.5
11	28.3	28.9	29.0	29.6	20.5	21.6	21.6	21.7	0.6	0.6	0.6	0.6	21.8	19.6	20.0	20.0	13.8	13.6	13.6	13.6
12	34.3	40.8	49.4	48.4	30.5	30.0	31.8	32.9	2.1	4.9	3.2	2.5	33.4	31.5	31.8	31.2	18.5	18.6	18.0	17.6
13	24.7	31.4	30.7	31.3	7.8	7.3	7.3	7.3	0.2	0.2	0.2	0.2	8.7	7.9	8.1	7.9	6.1	5.9	6.0	5.9
14	45.2	45.7	45.9	46.1	19.0	18.1	18.3	18.1	3.7	3.5	3.6	3.6	22.4	20.8	20.8	20.7	13.2	12.5	12.5	12.5
15	36.1	36.9	39.7	40.3	25.0	25.1	25.5	25.4	6.9	7.0	7.0	8.0	20.4	21.5	23.7	24.2	14.3	14.3	14.5	14.5
16	32.7	38.3	44.9	45.1	9.8	9.3	9.3	9.3	5.1	5.4	6.1	6.0	12.2	10.5	11.8	11.5	8.6	7.9	7.9	7.9
17	36.7	37.5	37.1	37.2	13.9	13.6	13.5	13.5	0.1	0.1	0.1	0.1	21.0	20.1	20.2	20.0	11.8	11.5	11.6	11.5
18	49.9	48.4	47.9	48.6	29.8	29.9	29.8	29.6	6.9	6.3	6.5	6.4	33.3	36.0	34.3	35.1	17.3	18.3	17.7	18.1
19	39.5	43.6	41.7	43.9	9.1	8.3	9.0	8.4	0.0	0.0	0.0	0.0	10.8	10.0	10.5	10.0	7.3	6.9	7.2	6.9
20	31.4	35.1	35.0	35.5	22.3	22.3	22.5	22.4	0.0	0.0	0.0	0.0	26.3	24.0	24.2	24.0	12.6	12.1	12.2	12.1
21	49.7	47.3	46.7	46.8	33.8	33.1	33.1	33.2	7.1	10.4	10.2	10.3	34.5	37.0*	35.6	35.6	16.9	18.8	18.7	18.7
22	47.7	44.9	46.7	43.4	14.7	17.4	17.3	15.9	3.7	3.8	5.6	4.2	26.3	25.2	25.0	24.9	15.6	15.4	15.3	15.3
23	41.9	40.3	40.4	39.6	10.0	10.8	11.1	11.0	0.0	0.0	0.0	0.0	10.7	9.2	9.2	9.4	6.9	6.6	6.6	6.6
24	45.8	45.9	44.7	46.9	28.0	27.9	28.2	28.2	1.3	1.4	1.4	1.8	35.2	35.4	34.2	34.1	17.3	18.2	17.6	17.1
25	47.9	47.8	48.9	49.3	16.0	16.2	16.3	14.7	0.0	0.0	0.0	0.0	4.3	4.2	4.1	3.8	3.1	3.1	3.1	2.9
26	46.0	46.4	46.6	46.4	25.2	24.6	25.1	24.7	0.0	0.0	0.0	0.0	12.4	11.1	10.6	10.4	7.2	6.7	6.6	6.5
27	47.5	49.0	48.0	48.7	24.7	25.1	25.0	24.8	0.0	0.0	0.0	0.0	20.8	19.2	20.2	18.8	11.0	10.6	10.8	10.5
28	33.0	36.1	42.2	39.4	21.1	20.4	21.9	20.7	0.9	0.8	0.8	0.8	20.1	17.9	18.4	17.4	12.9	11.8	11.8	11.5
29	45.8	48.8	49.9	49.6	27.7	31.4	32.9	32.2	1.3	1.3	1.4	1.2	34.8	36.7	36.8	35.4	15.4	16.4	16.5	16.2
30	43.2	48.3	46.0	46.3	30.4	30.3	29.9	30.1	3.6	4.6	4.3	4.9	35.8	33.2	35.8	31.6	18.1	17.9	18.6	17.5
31	35.3	38.3	37.5	38.0	18.7	16.6	16.8	16.7	0.1	0.0	0.0	0.0	31.7	27.3	28.7	27.5	20.0	18.9	19.3	19.0
32	32.6	35.0	36.0	36.1	23.5	24.8	25.8	25.2	0.0	0.3	0.1	0.6	25.7	22.9	21.3	20.9	13.2	12.6	12.2	12.2
33	33.4	38.5	36.6	39.0	32.2	32.2	32.0	32.4	0.7	0.8	0.8	0.8	12.2	13.5	13.3	13.8	11.6	11.2	11.2	11.1
34	47.3	47.6	48.1	47.5	23.6	24.0	23.7	23.8	0.0	0.0	0.0	0.0	20.1	17.8	19.4	18.3	11.2	10.9	11.1	10.9
35	48.2	48.6	47.9	48.1	29.2	28.5	29.0	29.0	0.0	0.0	0.0	0.0	17.3	19.1	17.8	16.6	8.3	9.0	8.5	8.5
36	38.1	42.6	43.1	44.4	20.6	19.5	18.0	18.0	7.2	10.1	9.9	11.7	34.7	31.7	30.1	29.7	20.2	19.7	18.8	18.5
37	36.5	37.5	37.2	38.9	20.6	19.5	20.7	20.6	3.2	3.2	3.4	3.5	20.0	20.3	19.3	19.0	11.9	11.8	11.5	11.4
38	29.8	33.2	31.0	34.2	14.5	14.0	14.1	15.2	2.8	3.1	3.3	3.6	26.3	26.2	26.3	24.2	15.2	14.8	15.0	14.4
39	46.2	48.0	46.7	47.1	19.3	19.5	19.2	19.6	0.0	0.0	0.0	0.0	6.8	5.6	6.3	6.6	4.5	4.3	4.5	4.5
40	22.8	22.3	22.6	30.3	5.5	6.2	5.2	6.7	0.0	0.0	0.0	0.0	15.1	14.9	15.3	14.1	9.7	9.9	9.7	9.3
41	32.1	32.9	31.2	33.4	19.3	18.6	19.0	18.7	1.6	2.3	2.1	2.1	21.1	18.4	18.1	17.4	12.8	11.3	11.0	10.8
42	40.8	47.9	47.5	50.1	23.4	22.8	22.6	22.7	20.8	29.0	29.2	34.7	36.5	32.6	30.2	28.4	17.4	16.2	15.8	15.5
43	22.0	25.1	22.2	21.9	4.2	3.2	4.0	3.6	2.1	2.1	2.2	2.2	19.2	20.3	19.9	19.1	11.5	11.4	11.5	11.0
44	32.0	32.8	32.3	36.3	20.0	20.0	19.8	19.9	17.9	17.8	17.6	17.6	27.3	25.2	23.0	22.6	16.9	16.1	15.9	15.8
45	44.1	44.5	42.9	45.1	26.5	27.1	27.9	27.1	0.0	0.0	0.0	0.0	23.5	20.5	23.0	20.7	12.5	11.8	12.3	11.8
46	41.7	43.7	46.1	45.8	21.8	22.2	21.7	22.0	5.1	5.5	5.7	5.6	25.2	23.4	23.7	22.3	13.7	13.3	13.2	12.9

Abbreviations: A plan, anatomical plan; V plan, ventilation-guided plan; Q plan, perfusion-guided plan; VQ plan, combined ventilation–perfusion-guided plan.

*Although the lung $V_{20\ Gy}$ value for patient 21 in the V plan is displayed as 37.0 % after rounding to one decimal place, the unrounded value was 36.96 %; therefore, the lung $V_{20\ Gy}$ planning constraint (< 37 %) was satisfied.

Table S4. Patient-level ROI-based dose-function metrics for high ventilated and high perfused lung across the four treatment plans.

Patient ID	High ventilated lung: Dmean (Gy)				High ventilated lung: $V_{5\ Gy}$ (%)				High ventilated lung: $V_{20\ Gy}$ (%)				High perfused lung: Dmean (Gy)				High perfused lung: $V_{5\ Gy}$ (%)				High perfused lung: $V_{20\ Gy}$ (%)			
	A plan	V plan	Q plan	VQ plan	A plan	V plan	Q plan	VQ plan	A plan	V plan	Q plan	VQ plan	A plan	V plan	Q plan	VQ plan	A plan	V plan	Q plan	VQ plan	A plan	V plan	Q plan	VQ plan
1	13.3	11.9	12.5	11.5	49.4	47.3	46.8	45.4	26.2	19.7	23.0	19.8	11.9	11.5	10.7	10.6	51.8	50.8	47.4	47.6	23.0	19.3	17.6	17.3
2	8.8	8.0	8.3	7.9	50.4	45.6	44.9	45.0	11.3	9.0	11.0	9.0	9.3	8.0	8.0	7.8	59.2	49.6	45.9	47.1	9.9	6.7	7.6	6.6
3	20.7	20.6	20.5	20.5	90.5	88.4	88.8	88.3	35.7	36.0	35.3	35.3	16.6	16.4	16.3	16.3	88.1	85.8	86.2	85.7	25.5	25.7	25.0	25.1
4	8.8	8.9	8.8	8.9	34.5	34.0	34.2	34.1	17.6	17.6	17.2	17.3	9.6	9.8	9.6	9.8	33.0	32.6	32.7	32.6	20.4	20.5	19.9	20.5
5	17.4	15.9	15.7	16.4	89.4	83.4	80.1	86.6	28.2	23.1	24.7	24.5	16.2	15.0	14.7	15.1	88.8	83.1	79.2	85.7	26.9	22.4	23.7	22.8
6	4.3	4.2	4.0	4.0	12.0	11.7	11.3	11.3	7.3	6.9	6.5	6.6	5.9	5.7	5.4	5.4	19.5	18.7	18.0	18.1	10.1	9.6	8.8	8.9
7	10.9	10.4	10.7	10.4	36.1	35.0	36.0	35.1	23.4	21.1	22.6	21.2	7.9	7.5	7.8	7.5	40.9	39.5	40.9	39.6	12.7	11.1	11.6	11.1
8	4.6	4.3	4.2	4.2	27.8	25.6	24.9	24.9	3.4	3.5	3.5	3.5	5.7	5.3	5.3	5.3	34.4	32.5	32.0	32.0	5.5	4.5	4.5	4.5
9	12.5	12.0	11.8	11.9	39.9	37.8	37.6	37.0	19.9	18.7	18.5	18.6	9.6	9.2	8.6	8.8	41.6	40.0	38.6	38.5	16.3	14.5	12.9	13.6
10	17.3	16.9	17.1	14.8	77.4	76.5	77.2	71.7	33.0	29.8	30.6	23.6	14.3	14.8	14.8	13.6	57.1	56.0	56.8	51.9	26.0	27.7	27.3	22.9
11	12.4	11.8	11.7	11.8	48.2	46.1	46.1	46.1	21.0	17.7	17.8	18.2	13.0	12.4	12.4	12.4	52.2	50.7	50.6	50.7	20.4	16.5	16.5	17.1
12	18.9	18.1	18.0	17.4	71.3	70.7	70.6	68.7	35.3	31.8	32.7	31.7	20.4	20.1	17.9	17.3	82.0	82.6	80.4	77.6	34.2	32.4	30.6	29.2
13	7.0	6.6	6.7	6.6	30.6	29.5	30.0	29.6	14.7	11.6	12.4	11.6	6.4	6.0	6.1	6.0	34.4	33.0	33.5	33.1	10.4	8.7	9.3	8.7
14	13.7	12.4	12.4	12.4	59.9	55.6	56.1	55.7	23.0	18.9	19.5	19.2	12.6	11.2	11.2	11.1	68.7	64.4	64.6	64.5	16.6	13.3	13.4	13.4
15	13.0	12.5	12.5	12.4	71.0	67.2	68.2	66.8	15.2	15.6	16.1	16.3	11.8	11.4	11.3	11.3	72.9	69.1	69.3	68.4	10.7	11.2	12.2	12.9
16	7.8	7.0	7.2	7.1	41.3	37.7	36.9	37.0	10.1	7.1	9.5	8.8	11.1	9.9	9.6	9.5	63.3	58.0	55.7	55.9	15.4	12.5	13.4	13.1
17	11.8	11.4	11.5	11.4	39.6	37.3	37.8	37.3	22.4	20.8	21.5	20.9	7.3	6.8	6.9	6.9	44.7	42.0	42.5	42.0	5.2	4.9	5.2	4.8
18	17.3	17.5	17.2	17.3	61.9	62.0	60.5	60.8	34.8	33.1	34.0	33.3	13.7	14.7	13.9	14.2	63.9	65.7	61.8	63.6	24.1	26.6	24.3	25.4
19	6.9	6.5	6.8	6.5	15.9	15.5	16.2	15.5	10.9	10.3	10.8	10.3	2.6	2.4	2.6	2.4	9.6	8.8	9.9	8.8	3.6	3.0	3.2	3.0
20	17.6	16.8	16.9	16.8	59.4	58.0	58.1	57.9	39.4	35.2	35.6	35.3	15.1	14.2	14.3	14.2	55.0	52.6	52.6	52.5	34.5	29.2	29.4	29.3
21	15.2	16.2	16.3	16.4	63.1	62.0	62.5	62.1	30.5	28.0	27.2	27.2	19.6	21.3	21.1	21.2	76.3	75.5	75.0	75.1	43.8	38.9	37.7	37.7
22	14.7	14.3	13.5	13.7	86.8	84.3	80.1	74.7	23.7	21.9	19.7	20.2	15.7	14.3	15.0	14.9	81.5	78.6	78.6	73.2	27.6	21.8	23.6	24.1
23	8.5	8.3	8.4	8.4	27.9	26.5	26.5	26.6	12.2	11.9	12.0	12.0	8.6	8.2	8.0	8.1	38.5	36.9	36.2	36.5	12.3	10.6	10.1	10.6
24	14.3	14.1	13.8	13.3	62.4	61.6	59.7	57.3	24.9	22.8	22.2	22.7	13.6	13.8	13.0	12.6	66.6	65.9	61.5	59.7	22.8	21.5	20.8	21.3
25	1.6	1.6	1.6	1.5	5.7	6.0	6.0	5.8	1.8	1.5	1.8	1.4	0.6	0.6	0.6	0.5	1.7	1.9	1.8	1.8	0.0	0.0	0.0	0.0
26	3.7	3.3	3.2	3.2	15.0	12.4	12.7	12.1	5.9	5.1	4.7	4.6	3.7	3.2	3.1	3.0	16.7	13.7	13.4	12.8	5.8	4.7	4.6	4.5
27	11.3	10.7	11.0	10.7	36.9	35.7	36.4	35.7	20.5	18.3	18.7	17.8	10.8	10.3	10.5	10.2	43.5	42.9	42.5	42.6	18.5	16.8	16.9	16.6
28	15.4	13.7	13.5	13.2	66.0	59.2	59.2	58.0	26.6	20.9	20.2	19.4	16.9	15.0	14.3	14.3	69.9	60.0	56.6	55.6	30.1	24.7	22.0	22.0
29	15.3	15.9	16.1	15.9	49.6	47.9	49.5	49.5	33.6	34.2	35.0	33.9	13.7	14.0	14.1	14.2	48.8	47.1	48.8	49.1	31.5	31.1	32.0	31.3
30	14.5	13.8	14.8	13.5	83.8	77.3	79.8	73.8	23.8	21.1	23.3	20.0	10.7	10.1	10.6	9.5	79.6	73.0	75.0	69.0	11.7	9.0	8.7	7.3
31	14.3	12.4	13.2	12.4	58.9	50.7	51.7	50.7	20.6	16.8	18.7	16.9	15.0	12.9	13.4	13.0	68.8	63.1	63.7	63.2	19.5	12.9	14.0	13.0
32	7.6	6.8	6.9	6.7	26.2	23.1	24.3	22.4	12.1	10.0	10.2	9.6	8.9	8.2	7.2	7.2	38.3	36.3	35.3	34.6	13.6	11.6	9.0	9.0
33	11.9	11.6	11.0	10.8	79.6	67.3	65.1	62.7	10.8	14.0	13.7	14.2	12.4	11.2	11.4	11.3	76.6	67.0	63.9	63.0	12.3	13.3	13.5	14.1
34	5.8	5.5	5.7	5.5	25.1	23.4	23.2	22.6	7.9	6.5	8.0	6.7	6.2	5.7	5.8	5.6	32.1	30.1	29.5	28.6	7.1	4.5	6.2	5.2
35	7.3	6.9	6.8	6.8	25.9	24.5	24.0	23.4	15.7	13.6	13.2	13.1	3.2	3.7	3.2	3.2	18.3	18.7	17.8	17.1	3.3	5.7	3.4	3.5
36	21.5	17.0	17.8	15.6	83.1	71.2	62.8	59.2	40.5	27.2	31.0	24.9	17.6	14.5	13.5	12.3	82.6	73.0	56.8	57.4	29.8	20.8	19.6	17.3
37	10.9	9.9	10.2	9.8	53.9	45.6	49.1	45.4	16.2	12.9	14.8	12.9	8.6	7.9	7.4	7.2	53.9	44.5	46.3	43.8	9.1	9.4	6.2	6.2
38	16.0	14.3	15.3	14.1	56.7	47.2	50.9	48.1	25.8	22.1	24.8	22.2	14.2	13.0	13.1	12.6	54.6	46.9	45.5	47.2	22.3	20.8	19.6	18.7
39	2.2	2.1	2.3	2.1	10.3	10.2	10.9	10.3	2.4	1.6	2.3	2.0	2.0	1.9	2.0	2.0	10.2	10.4	10.9	10.5	1.5	1.0	1.1	1.3
40	19.0	18.8	19.1	17.6	54.4	54.0	54.1	52.5	34.8	33.7	34.9	30.9	10.6	10.9	9.9	10.4	55.1	56.0	49.0	50.8	15.2	15.1	14.3	15.6
41	14.5	11.8	11.7	11.6	64.1	46.0	43.9	43.1	24.4	19.0	19.5	18.9	14.7	12.0	10.6	10.6	72.5	56.7	45.4	45.4	24.7	18.9	17.4	17.1
42	21.1	18.2	19.3	18.3	75.1	63.2	64.9	61.9	45.6	34.6	38.4	33.9	14.1	10.9	10.0	9.4	68.8	56.4	52.6	52.2	29.0	20.9	14.8	12.9
43	13.5	13.0	13.1	12.2	64.2	51.7	58.1	49.1	22.5	23.4	23.2	21.3	9.4	9.2	9.2	8.8	44.4	35.9	39.6	35.0	16.7	17.4	16.6	16.2
44	19.3	18.0	18.2	17.9	72.7	65.8	68.7	65.9	34.5	30.0	30.5	29.8	13.4	11.8	12.1	11.8	71.1	61.3	64.6	62.9	16.5	12.8	11.8	11.7
45	13.4	11.9	12.6	11.9	44.2	37.6	40.2	38.0	22.4	19.0	20.8	19.0	12.4	10.9	11.1	10.7	48.8	42.9	44.3	42.8	22.5	18.0	18.0	17.3
46	15.2	14.1	14.3	13.8	76.8	68.6	68.6	67.5	27.4	22.9	25.7	23.0	13.5	13.0	11.8	11.6	70.1	65.8	59.6	59.8	23.5	22.5	17.5	17.3

Abbreviations: A plan, anatomical plan; V plan, ventilation-guided plan; Q plan, perfusion-guided plan; VQ plan, combined ventilation–perfusion-guided plan; ROI, region of interest; MLD, mean lung dose.

Table S5. Patient-level voxel-based ventilation- and perfusion-weighted dose-function metrics across the four treatment plans.

Patient ID	Ventilation-based fMLD (Gy)				Ventilation-based $fV_{5\ Gy}$ (%)				Ventilation-based $fV_{20\ Gy}$ (%)				Perfusion-based fMLD (Gy)				Perfusion-based $fV_{5\ Gy}$ (%)				Perfusion-based $fV_{20\ Gy}$ (%)			
	A plan	V plan	Q plan	VQ plan	A plan	V plan	Q plan	VQ plan	A plan	V plan	Q plan	VQ plan	A plan	V plan	Q plan	VQ plan	A plan	V plan	Q plan	VQ plan	A plan	V plan	Q plan	VQ plan
1	12.2	11.8	11.8	11.5	61.7	61.6	60.3	59.9	27.8	25.1	25.9	24.9	12.2	12.1	11.6	11.6	61.4	61.4	58.9	59.1	25.1	23.8	22.9	23.0
2	8.7	8.2	8.4	8.1	54.2	49.0	47.4	47.9	12.8	11.0	12.5	11.1	9.1	8.4	8.5	8.3	57.0	50.3	47.8	48.6	12.6	10.6	11.6	10.6
3	15.5	14.4	15.1	14.4	84.5	77.5	79.5	76.8	33.9	30.7	33.1	30.8	16.2	15.3	15.7	15.3	85.2	77.1	78.7	76.4	30.8	28.9	30.3	29.0
4	9.5	9.7	9.6	9.7	39.2	39.0	39.1	39.0	24.6	24.7	23.8	24.6	9.2	9.3	9.2	9.4	37.0	36.7	36.8	36.7	23.5	23.5	22.7	23.5
5	15.5	14.8	14.6	15.1	91.8	88.0	85.5	89.7	33.3	30.6	30.8	32.2	16.3	15.8	15.4	16.0	90.9	87.0	84.3	88.7	32.7	31.4	30.4	31.7
6	7.8	7.5	7.3	7.3	29.7	29.1	28.4	28.5	17.3	15.4	15.0	15.0	7.4	7.1	6.9	6.9	28.8	28.0	27.2	27.3	15.6	14.1	13.6	13.6
7	11.2	10.9	11.1	10.9	45.5	44.5	45.5	44.5	23.5	21.9	22.8	21.9	9.9	9.5	9.8	9.5	46.5	45.3	46.5	45.3	19.1	17.8	18.4	17.8
8	7.4	7.3	7.2	7.2	37.7	36.4	35.8	35.8	13.3	13.2	13.1	13.1	6.9	6.7	6.6	6.6	36.2	35.0	34.5	34.5	11.5	11.1	11.0	11.0
9	12.4	11.9	11.6	11.7	48.1	46.4	45.5	45.4	23.6	21.4	20.5	20.6	11.4	11.0	10.6	10.7	48.1	46.6	45.4	45.3	22.1	19.6	18.4	18.6
10	12.3	13.2	13.3	12.6	65.1	65.0	64.0	62.5	28.8	32.3	33.5	29.4	15.2	15.9	16.0	15.0	74.0	73.8	73.0	70.9	33.4	34.8	36.2	31.9
11	13.0	12.7	12.7	12.7	56.4	55.2	55.1	55.1	22.1	19.5	19.8	20.0	12.5	12.2	12.2	12.2	53.8	52.6	52.5	52.6	21.0	17.9	18.2	18.4
12	17.9	17.8	17.2	16.8	75.8	76.1	74.9	72.5	35.0	32.5	33.1	32.2	18.5	18.5	17.3	16.8	79.5	80.1	78.4	75.8	35.1	33.1	32.8	31.8
13	6.2	6.0	6.1	6.0	34.6	33.5	33.9	33.5	11.1	9.5	9.9	9.5	6.1	5.8	5.9	5.8	35.7	34.5	34.9	34.5	9.7	8.5	8.9	8.5
14	12.8	11.9	12.0	11.9	64.5	61.0	61.4	61.2	23.3	20.9	21.1	20.9	12.4	11.4	11.4	11.4	67.2	63.7	63.9	63.8	20.7	18.4	18.4	18.3
15	10.9	10.7	10.8	10.8	71.0	68.5	69.0	68.1	18.0	18.8	20.5	21.0	12.0	11.8	11.9	11.9	70.1	67.8	68.0	67.3	15.9	16.7	18.7	19.3
16	8.0	7.3	7.3	7.3	45.4	42.0	40.9	41.0	12.1	9.8	11.7	11.2	9.2	8.3	8.2	8.1	53.8	49.7	48.0	48.1	13.9	11.6	12.8	12.6
17	11.3	11.0	11.0	11.0	45.5	43.4	43.8	43.4	21.7	20.6	20.9	20.6	8.3	7.9	8.0	7.9	43.3	41.0	41.5	41.0	11.4	10.8	11.0	10.8
18	16.1	16.9	16.4	16.7	61.4	62.0	60.4	60.9	34.0	35.5	34.4	34.7	13.9	14.8	14.1	14.4	60.4	61.7	59.3	60.2	27.1	30.0	28.1	28.8
19	7.0	6.7	6.9	6.7	23.3	22.8	23.9	22.8	11.6	10.9	11.5	10.9	4.9	4.7	4.9	4.7	18.5	17.8	19.1	17.8	8.1	7.4	7.9	7.4
20	14.1	13.5	13.6	13.5	55.8	53.9	54.0	53.8	32.5	29.1	29.4	29.2	13.4	12.7	12.8	12.7	53.7	51.5	51.6	51.4	31.0	27.1	27.3	27.1
21	15.2	16.9	16.8	16.8	70.3	69.4	69.8	69.4	33.7	34.4	33.3	33.3	17.1	19.0	18.9	18.9	76.2	75.4	75.4	75.1	39.6	39.8	38.5	38.4
22	12.8	12.3	12.4	12.4	79.9	78.0	76.1	72.8	25.6	22.7	23.3	23.6	14.8	14.6	14.2	14.2	82.0	80.1	76.9	73.5	24.7	23.3	22.4	22.6
23	7.4	7.2	7.2	7.2	30.8	29.7	29.5	29.6	11.9	10.8	10.8	10.9	7.4	7.1	7.0	7.1	33.6	32.4	32.0	32.1	11.8	10.2	10.0	10.3
24	15.3	15.9	15.5	14.9	67.6	69.8	65.9	64.4	32.6	32.7	31.9	30.7	15.1	15.9	15.3	14.5	69.5	71.8	67.4	65.7	32.2	32.4	31.5	30.4
25	2.6	2.6	2.6	2.4	10.7	11.1	10.9	10.5	3.7	3.6	3.5	3.2	1.8	1.8	1.8	1.7	7.6	7.9	7.8	7.5	2.1	2.1	2.1	1.9
26	6.0	5.5	5.5	5.4	28.4	25.5	25.9	25.2	11.4	10.0	9.6	9.3	5.5	4.9	4.9	4.8	26.7	23.7	24.0	23.3	10.0	8.5	8.2	8.0
27	10.6	10.2	10.4	10.1	43.4	42.2	42.6	42.2	21.8	20.0	20.8	19.6	10.4	10.0	10.2	9.9	45.4	44.4	44.5	44.2	21.0	19.6	20.1	19.0
28	12.2	11.0	10.8	10.6	63.3	54.8	53.4	51.7	24.1	20.4	20.4	19.3	14.6	13.1	12.8	12.5	64.2	55.1	52.7	51.0	24.3	20.5	19.5	19.0
29	14.8	15.5	15.7	15.4	53.7	52.7	53.7	53.8	36.2	37.3	37.8	36.4	14.2	14.9	15.0	14.8	51.7	50.5	51.7	52.0	34.8	35.4	36.0	35.0
30	15.9	15.5	16.4	15.2	84.9	80.8	82.1	78.3	33.7	30.5	33.5	29.0	13.3	12.9	13.5	12.5	81.0	76.6	77.8	73.9	25.3	22.6	23.9	20.9
31	16.8	15.4	15.9	15.5	67.0	61.9	62.7	62.0	26.7	22.3	23.9	22.5	15.5	14.0	14.4	14.0	68.6	63.8	64.4	63.8	23.6	18.1	19.5	18.3
32	10.9	10.2	10.0	9.8	44.0	41.8	42.8	41.6	21.7	18.9	17.8	17.2	10.9	10.2	9.7	9.6	46.3	44.6	44.7	44.1	21.8	18.8	16.6	16.2
33	9.4	8.8	8.9	8.8	76.8	67.2	65.6	64.2	11.2	13.0	13.2	13.5	11.3	10.8	10.7	10.6	75.9	68.5	66.0	65.2	11.7	13.1	12.9	13.3
34	8.5	8.2	8.4	8.2	36.9	36.0	35.6	34.9	14.5	12.6	14.2	13.1	7.3	6.9	7.1	6.9	35.8	34.7	34.2	33.4	11.5	9.3	11.0	9.9
35	7.7	8.0	7.7	7.6	32.4	32.8	32.3	31.8	17.0	17.7	15.7	15.6	5.6	6.2	5.7	5.7	27.5	28.4	27.7	27.1	10.9	12.9	10.5	10.5
36	16.9	15.2	15.0	14.1	82.4	79.5	70.8	69.2	37.0	29.4	30.1	27.2	18.2	16.8	15.8	15.2	82.6	79.3	68.3	67.9	32.3	27.1	25.4	24.4
37	11.0	10.6	10.4	10.2	58.3	53.3	54.9	52.9	19.3	18.4	18.2	17.2	9.6	9.3	9.0	8.8	56.7	51.0	52.3	50.4	14.8	15.0	13.7	13.1
38	14.6	13.7	14.1	13.4	58.7	52.6	54.1	52.3	27.1	25.5	26.4	24.1	14.0	13.3	13.5	12.9	58.7	52.8	52.5	52.5	26.0	25.1	24.6	22.8
39	3.6	3.5	3.7	3.6	18.7	18.9	19.4	19.0	5.8	4.5	5.2	5.4	3.3	3.2	3.3	3.3	17.5	17.7	18.2	17.8	4.9	3.7	4.3	4.5
40	10.3	10.4	10.3	9.7	50.6	50.5	49.3	46.6	22.5	21.9	22.6	20.3	8.1	8.3	7.9	7.9	62.0	62.4	58.3	57.6	20.4	20.3	20.2	19.8
41	12.4	10.5	10.2	10.1	62.0	47.4	44.5	43.8	22.4	18.3	18.2	17.6	12.7	10.8	10.2	10.1	67.2	52.9	47.1	46.6	22.8	18.6	18.1	17.5
42	18.8	16.9	17.1	16.5	71.2	63.2	62.6	61.4	40.1	33.5	33.1	30.4	15.2	13.4	12.9	12.5	66.8	58.1	55.7	55.3	31.1	26.5	22.5	21.0
43	11.8	11.5	11.6	11.0	54.2	46.7	50.6	45.1	20.2	21.2	20.9	19.6	10.4	10.2	10.2	9.8	46.3	40.0	43.0	39.0	18.7	19.5	18.9	18.3
44	16.5	15.5	15.5	15.3	70.5	65.0	67.1	65.2	29.1	25.7	24.6	24.1	13.4	12.3	12.3	12.1	68.4	61.8	64.2	62.5	20.0	17.0	15.4	14.9
45	13.6	12.6	13.2	12.5	51.6	47.2	49.1	47.6	25.2	21.7	24.2	21.8	12.7	11.7	12.1	11.6	51.4	47.0	48.6	47.2	23.9	20.1	21.9	19.9
46	14.5	13.8	13.8	13.5	71.5	66.8	65.6	65.2	26.9	24.0	25.3	23.5	13.7	13.2	12.7	12.4	68.5	64.9	61.7	61.6	24.8	23.0	21.6	20.5

Abbreviations: A plan, anatomical plan; V plan, ventilation-guided plan; Q plan, perfusion-guided plan; VQ plan, combined ventilation–perfusion-guided plan; fMLD, mean lung dose weighted by regional function; $fV_{5\ Gy}$ and $fV_{20\ Gy}$, percent lung function receiving 5 and 20 Gy, respectively.
